# Taxonomic distribution of large DNA viruses in the sea

**DOI:** 10.1186/gb-2008-9-7-r106

**Published:** 2008-07-03

**Authors:** Adam Monier, Jean-Michel Claverie, Hiroyuki Ogata

**Affiliations:** 1 Structural and Genomic Information Laboratory, CNRS-UPR 2589, IFR-88, Université de la Méditerranée Parc Scientifique de Luminy, avenue de Luminy, FR-13288 Marseille, France

## Abstract

**Background:**

Viruses are ubiquitous and the most abundant biological entities in marine environments. Metagenomics studies are increasingly revealing the huge genetic diversity of marine viruses. In this study, we used a new approach - 'phylogenetic mapping' - to obtain a comprehensive picture of the taxonomic distribution of large DNA viruses represented in the Sorcerer II Global Ocean Sampling Expedition metagenomic data set.

**Results:**

Using DNA polymerase genes as a taxonomic marker, we identified 811 homologous sequences of likely viral origin. As expected, most of these sequences corresponded to phages. Interestingly, the second largest viral group corresponded to that containing mimivirus and three related algal viruses. We also identified several DNA polymerase homologs closely related to Asfarviridae, a viral family poorly represented among isolated viruses and, until now, limited to terrestrial animal hosts. Finally, our approach allowed the identification of a new combination of genes in 'viral-like' sequences.

**Conclusion:**

Albeit only recently discovered, giant viruses of the Mimiviridae family appear to constitute a diverse, quantitatively important and ubiquitous component of the population of large eukaryotic DNA viruses in the sea.

## Abstract 2

Phylogenetic mapping of metagenomics data reveals the taxonomic distribution of large DNA viruses in the sea, including giant viruses of the Mimiviridae family.

## Background

Viruses are ubiquitous and the most numerous microbes in marine environments. Previous analyses using electron microscopy, epifluorescence microscopy and flow cytometry revealed the existence of 10^6 ^to 10^9 ^virus-like particles per milliliter of sea water [1-3]. Infecting marine organisms from oxygen-producing phytoplankton to whales, viruses regulate the population of many sea organisms and are important effectors of global biogeochemical fluxes [4,5]. It is also becoming clear that viruses hold a great genetic diversity; comparative genomics [6,7] and virus-targeted metagenomics studies [8-10] revealed a large amount of viral sequences having no detectable homologs in the databases. As a reservoir of 'new' genes as well as vectors of 'old' genes, viruses may significantly contribute to the evolution of microorganisms in marine ecosystems.

Despite this progress in characterizing the environmental significance of viruses, a quantitative description of the marine virosphere remains to be done. This includes the determination of the relative abundance of virus families and the assessment of the level of their genetic diversity. In this context, large viruses, whose particle sizes can exceed those of small bacteria [11], are of particular concern. Most of them, such as *Acanthamoeba polyphaga *[12], may be retained on the 0.16-0.2 μpore filters specifically used in virus-targeted metagenomic studies and may not be gathered in the fraction traditionally associated with viral sequences [11]. A recently released marine microbial metagenomic sequence data set, produced by the first phase of the Sorcerer II Global Ocean Sampling (GOS) Expedition [13], provides an opportunity to quantitatively investigate viral diversity in marine environments. The GOS data comprise a large environmental shotgun sequence collection, with 7.7 million sequencing reads assembled into 4.9 billion bp contigs. In the GOS expedition, microbial samples were collected mainly from surface sea waters, and some others were collected from non-marine aquatic environments. Most DNA samples were extracted from the 0.1-0.8 μsized fraction, which is dominated by bacteria. Williamson *et al*. [14] recently reported that at least 3% of the predicted proteins contained within the GOS data are of viral origin. Notably, a number of sequences most similar to the genome of the giant mimivirus have been found in the Sargasso Sea metagenomic data set [15], produced by a pilot study of the GOS expedition [16], as well as in the new GOS metagenomic data set [17].

Determining taxonomic distribution, referred to as 'binning', is the first step to analyze microbial populations in metagenomic sequences [18]. One simple binning approach uses database search programs such as BLAST to find the best scoring sequence of known species. A majority rule can be used to assign a taxonomic group to a metagenomic sequence [14,19]. Similar to the best hit criterion used to define orthologous genes in complete genomes [20,21], two-way BLAST searches were used to detect 'mimivirus-like' sequences in metagenomic data [15,17]. Such a post-processing of homology search results can improve the accuracy of taxonomic assignment. However, the use of homology search programs has serious drawbacks [22]. For instance, BLAST scores are highly sensitive to alignment sizes and to insertions/deletions. Further, it is difficult to infer evolutionary distances among high scoring hits only from the BLAST scores.

Phylogenetic analysis remains the most powerful way to determine taxonomic distribution of metagenomic sequences. Short and Suttle [23] used phylogenetic methods to classify PCR-amplified gene sequences and suggested the existence of previously unknown algal viruses in coastal waters. Similar phylogenetic studies were performed to assess the diversity of T4-type phages [24] or RNA viruses [25,26] in marine environments. In these studies, different markers, such as the major capsid genes or RNA-dependent RNA polymerase gene sequences, were amplified by PCR or RT-PCR and analyzed by phylogenetic methods. To examine taxonomic distribution of large DNA viruses in a metagenomic sequence collection, B-family DNA polymerase (PolB) is a useful marker [23,27,28]. PolB sequences are conserved in all known members of nucleocytoplasmic large DNA viruses (NCLDVs) [29], which include 'Mimiviridae' [30], Phycodnaviridae, Iridoviridae, Asfarviridae, and Poxviridae. PolB genes are also found in other eukaryotic viruses, such as herpesviruses, baculoviruses, ascoviruses and nimaviruses, in some bacteriophages (for example, T4-phage, cyanophage P-SSM2), and in some archaeal viruses (for example, Halovirus HF1). Eukaryotes have four PolB paralogs (catalytic subunits of α, δ, ε and ζ DNA polymerases). PolB genes are found in all of the main archaeal lineages (Nanoarchaeota, Crenarchaeota and Euryarchaeota). The presence of PolB homologs in bacteria (the prototype being *Escherichia coli *DNA polymerase II) is limited; PolBs are found in Proteobacteria, Acidobacteria, Firmicutes, Chlorobi and Bacteroidetes. PolB genes are suitable for the classification of large DNA viruses [31,32] thanks to their strong sequence conservation and an apparently low frequency of recent horizontal transfer [28,33].

When applying phylogenetic methods to environmental shotgun sequences, the treatment of short sequences requires special attention. These sequences show large variation in size and possibly correspond to different parts of a selected marker gene. Piling up multiple short sequences on representative markers from known organisms does not provide an appropriate alignment (whatever software is used) with enough signals for the subsequent phylogenetic analysis. In this study we developed a new phylogeny-based method. The method called 'phylogenetic mapping' analyzes individual metagenomic sequences one by one and determines their phylogenetic positions using a reference multiple sequence alignment (MSA) and a reference tree. As an attempt to investigate the presence, the taxonomic richness and the relative abundance of different large DNA viruses in marine environments, we analyzed the GOS data set using PolB sequences as our reference. Our study does not address the abundances of small DNA viruses or RNA viruses [14,34].

## Results

### Phylogenetic mapping

**Figure 1:**
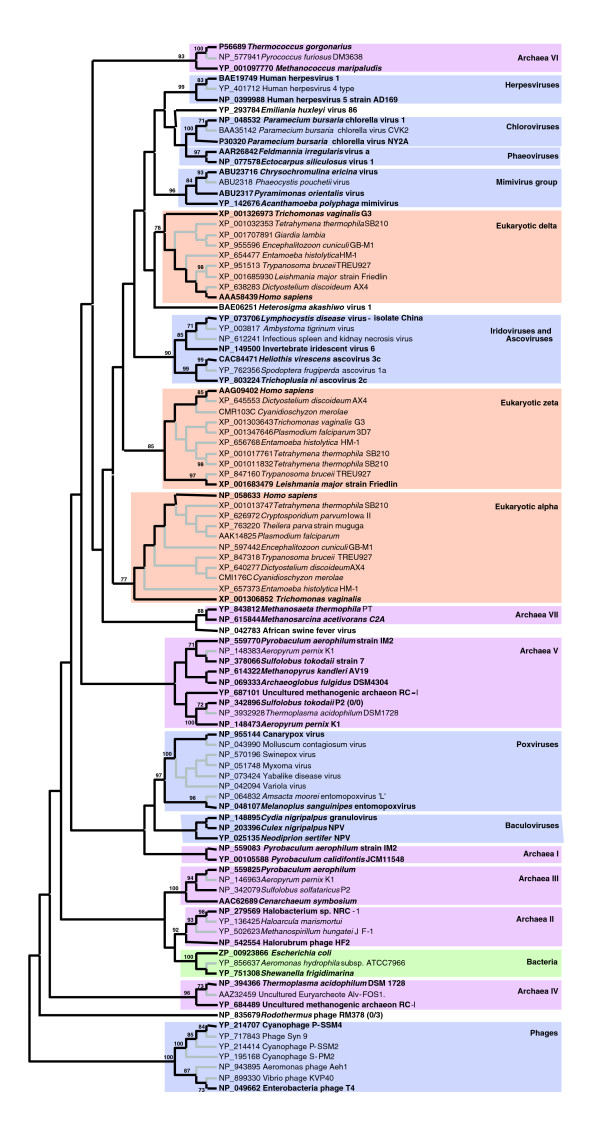
Maximum likelihood tree of 101 PolB sequences in the complete reference set. The phylogenetic tree was built using PhyML [73] (Jones-Taylor-Thornton substitution model [76], 100 bootstrap replicates) based on a multiple sequence alignment generated using M-Coffee [72]. This tree is unrooted *per se*. The phage group was arbitrarily chosen as an outgroup for presentation purposes. The lengths of branches do not represent sequence divergence. Bootstrap values lower than 70% are not shown. The selected 51 representatives for the phylogenetic mapping and the associated branches are highlighted in bold face and black lines, respectively. Different colors correspond to different taxa: viruses (blue), eukaryotes (orange), bacteria (green) and archaea (pink).

We searched the GOS data set for PolB-like sequences using the Pfam hidden Markov profile (PF00136). This resulted in a set of 1,947 sequences (from 23-562 amino acid residues). These sequences are referred to as 'PolB fragments' in this study. We next built a reference MSA of PolB homologs from known organisms (Additional data file 1). The reference MSA (Additional data file 2) corresponds to the polymerase domains of PolB homologs and contains 101 sequences, which were selected to achieve the widest possible taxonomic/paralog coverage (but with a non-exhaustive sampling for closely related species) for the analysis of the GOS metagenomic data. The reference MSA was used to generate a maximum likelihood tree (that is, the reference tree; Figure 1). Although the phylogenetic reconstruction did not provide statistical support for most of the basal branches, many peripheral groupings (supported by bootstrap values ≥ 70%) were coherent with the current taxonomy of viruses and cellular organisms. In this tree, we identified eight viral groups: poxviruses; chloroviruses; phaeoviruses; mimivirus and related algal viruses (*Pyramimonas orientalis *virus PoV01, *Chrysochromulina ericina *virus CeV01 and *Phaeocystis pouchetii* virus PpV01); iridoviruses grouped with ascoviruses; herpesviruses; baculoviruses; and one phage group. The PolB homologs from African swine fever virus (ASFV, Asfarviridae), *Emiliania huxleyi *virus 86 (EhV-86, Phycodnaviridae), *Heterosigma akashiwo* virus 1 (HaV, Phycodnaviridae) and the phage RM378 did not show well supported clustering with other PolB sequences. We also identified eleven groups in the reference tree for cellular PolB homologs: seven archaeal groups, one bacterial group and three eukaryotic groups (α, δ and ζ subtypes). Each of the GOS PolB fragments was then examined for its phylogenetic position using the reference MSA and the reference tree. To reduce the computation time and to streamline tprocess of summarizing results, we reduced the size of the reference MSA. Specifically, we selected 51 representatives from the 101 reference sequences and removed the remaining sequences. The reference tree was also reduced so that the resulting tree contains only the selected 51 representatives, while we conserved the original topology of the full reference tree shown in Figure 1. The reduced reference tree has 99 branches (including internal branches). A constraint on this topology defines 99 possible branching positions for each of the GOS PolB fragments. We aligned, one by one, each of the PolB fragments on the reduced reference MSA using the T-Coffee profile method. Based on the resulting profile MSA containing 52 sequences, the likelihoods for all 99 possible branching positions (thus 99 different topologies) were computed by ProtML [35]. A statistical significance for the best tree among the 99 topologies was assessed by the RELL (resampling of estimated log likelihoods) bootstrap method [36,37]. We considered the branching position of a PolB fragment to be supported when the RELL bootstrap value for the best topology was ≥ 75%.

### Diversity of large DNA viruses in the GOS data set

**Figure 2:**
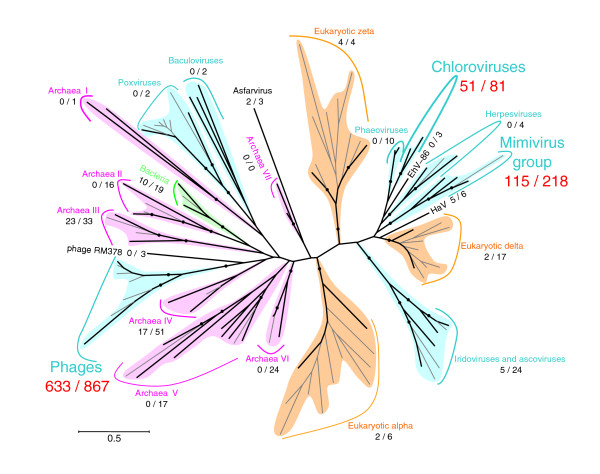
Phylogenetic mapping results of the GOS PolB fragments. Results of the phylogenetic mapping are summarized and displayed for each group in the reference tree. Numbers in parentheses (*X*/*Y*) are the total number of mapped PolB fragments (*Y*) and the number of supported cases (*X*). The tree topology is the same as the one shown in Figure 1. Branches with bootstrap values ≥ 70% are marked with filled circles. The 99 branches examined by our phylogenetic mapping are shown with black lines; other peripheral branches are shown with gray lines. The length of the scale bar corresponds to 0.5 substitutions per site. colors correspond to different taxa: viruses (blue), eukaryotes (orange), bacteria (green) and archaea (pink).

Our phylogenetic mapping method could assign the best branching position for 1,423 PolB fragments, of which 1,224 (86%) were mapped on viral branches. The best branching position was statistically supported by the RELL method for 869 PolB fragments, of which 811 (93%) were mapped on viral branches. Figure 2 and Additional data file 3 show the taxonomic distribution of the GOS PolB fragments. The largest fraction of the PolB fragments was mapped on the phage group. Of 866 cases of mapping within the phage group, 633 were supported. This appears consistent with the current estimate of the large number of phage-like particles and their genetic richness in marine environments [3]. The second largest number of supported mappings was found to fall into large eukaryotic viruses commonly found in aquatic environments. Among them, the 'Mimiviridae group' (mimivirus, PoV01 and CeV01 [17]) represented the largest fraction, with 115 supported cases. The chlorovirus group gathered 51 supported cases of mapping. The iridovirus/ascovirus group and the branch leading to HaV showed five supported mappings each. In contrast, no PolB fragment was mapped for the groups for baculoviruses or herpesviruses commonly found in terrestrial animals. Interestingly, we found two PolB fragments mapped with good support on the ASFV branch (JCVI SCAF 1101668126451, JCVI SCAF 1101668152950). When these two PolB fragments were compared to the NCBI non-redundant amino acid sequence database (NRDB) using BLASTP, they were most similar to the ASFV PolB sequence. ASFV is pathogenic to domestic pigs and is currently the sole representative of the Asfarviridae family [38]. Concerning cellular organisms, eukaryotic homologs gathered few mappings, as expected from the sample filtration threshold used in the GOS metagenomic study. Two archaeal groups - the group III containing crenarchaeotes (for example, *Pyrobaculum aerophilum, Cenarchaeum symbiosum*) and the group IV containing euryarchaeotes (for example, *Thermoplasma acidophilum*, an uncultured euryarchaeote Alv-FOS1) - had 23 and 17 supported cases of mapping, respectively. The bacterial group presented ten supported mappings.

### Validation of the mapping results using long PolB fragments

**Figure 3:**
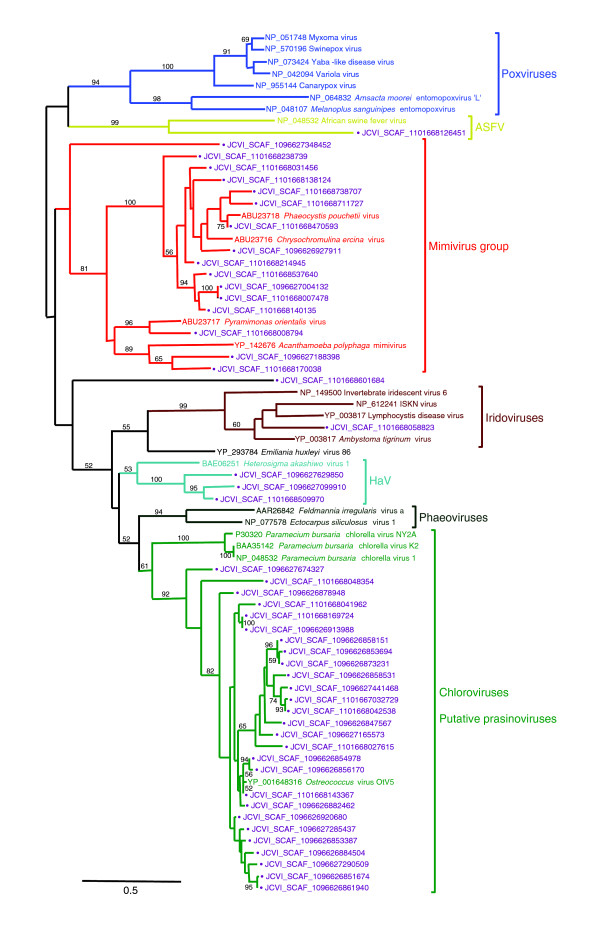
Maximum likelihood tree of PolB sequences belonging to NCLDVs. The phylogenetic tree was built using PhyML [73] (Jones-Taylor-Thornton substitution model [76], 100 bootstrap replicates) based on a multiple sequence alignment generated using MUSCLE [77]. Bootstrap values lower than 50% are not shown. GOS sequences are marked with filled circles and displayed in purple. The tree was mid-point rooted. The DNA polymerase gene from the recently released *Ostreococcus *virus OtV5 (GenBank: EU304328) was included in this tree. The OtV5 PolB was not included in our reference set as it was not available at the time of our phylogenetic mapping study. The length of the scale bar corresponds to 0.5 substitutions per site.

We examined the phylogenetic mapping result and the sequence diversity of the PolB fragments classified in large eukaryotic virus groups (that is, NCLDVs). From those mapped on NCLDV branches, we selected long PolB fragments that generated a profile MSA showing at least 150 non-gapped sites. We computed a single alignment of these long PolB fragments together with the reference PolB sequences from large eukaryotic virus groups. A maximum likelihood tree (Figure 3) based on the alignment was perfectly consistent with our one-by-one mapping result (Figure 2) in terms of taxonomic assignment. The Mimiviridae group contained 16 PolB fragments showing substantial sequence variations. Twelve of them were significantly closer (bootstrap 100%) to CeV01 or PpV01 (both viruses of haptophytes) than to mimivirus or PoV01 (a green algal virus). Three of the rest were grouped with either mimivirus (bootstrap 89%) or PoV01 (bootstrap 96%). The last one (JCVI SCAF 1096627348452) was placed at the basal position of the Mimiviridae group. Although this basal positioning was not statistically supported, it was consistent with our one-by-one phylogenetic mapping result. The mimivirus PolB shared 47% identical amino acid residues with its closest homolog (JCVI SCAF 1101668170038). A large and diverse group containing 27 PolB fragments (bootstrap 92%) was also found beside the chlorella virus group (*Paramecium bursaria *chlorella viruses 1, K2 and NY2A). The DNA polymerase gene from the recently released *Ostreococcus *virus OtV5 genome (GenBank: EU304328) [39] was found grouped together with these PolB fragments. The grouping of a PolB fragment with ASFV PolB was also confirmed (bootstrap 100%).

### Viral PolBs are more diverse than bacterial PolBs

We investigated the abundance of viral PolB genes relative to bacterial PolB genes in the GOS data set. Here, we used read coverage as a proxy to measure the abundance of the cognate DNA molecules in the samples. We computed the read coverage of each contig harboring a PolB fragment mapped on the reference tree with significant support, and then obtained the median of the read coverage values for each set of contigs mapped on the same branch (Additional data file 3). PolB sequences mapped on viral branches exhibited low median coverage values ranging from 1.31 for the ASFV branch to 2.00 for a phage branch. The median coverage value for the contigs mapped on the mimivirus branch (12 contigs) was 1.32. The viral contig with the largest read coverage (6.68) was the one mapped on the cyanophage P-SSM4 branch. In contrast, a higher median coverage value (8.40) was found for bacterial contigs mapped on the branch leading to *Shewanella frigidimarina*. One of the bacterial contigs exhibited a read coverage of 29.17. Viral branches were thus characterized by a large number of mapped contigs exhibiting a low coverage. This is consistent with numerous and very diverse viral populations [40]. On the other hand, the bacterial branches exhibited a lower number of mapped contigs with a larger read coverage. This is consistent with numerous but less diverse populations of bacterial species, although our results concern only bacteria having PolB homologs.

### Geographic distributions of viral PolBs

**Figure 4:**
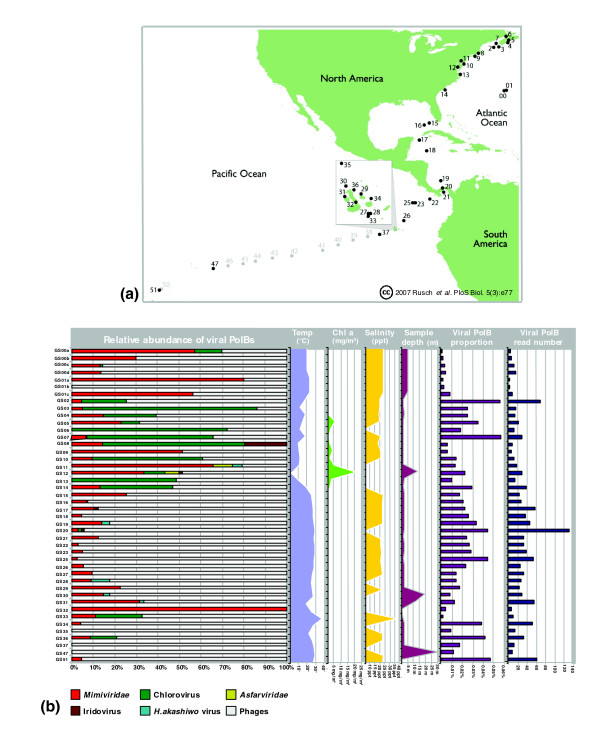
Geographic localization. **(a) **The different sampling sites of the Sorcerer II Global Sampling expedition. The samples 00 and 01 are part of the Sargasso Sea pilot study [16]. The inset shows samples 27 to 36, which were sampled in the Galapagos Islands. The sampling sites displayed in light gray were not analyzed in the GOS original study, nor in this study. This part of Figure 1 was reproduced from [13]. **(b) **Relative abundance of PolB fragments for virus groups across GOS sampling sites. The left-most panel shows the relative abundance of viral PolBs in difierent GOS samples. The mimivirus group clearly appears as the most ubiquitous after phages. Four area plots (second to fifth panels from the left) show water temperature, chlorophyll *a *concentration (no information was available for GS20, GS30, GS32, GS33, GS47 and GS51 sites), salinity (no information was available for GS06, GS11, GS13, GS14, GS28, GS30, GS31, GS32, GS34 and GS37 sites) and sample depth, respectively. Two far right histograms (sixth and seventh panels) show the proportion and the estimated number of reads associated with the viral PolB fragments among total reads for a given sample.

GOS metadata provide physicochemical and biological parameters associated with each sampling site, such as water temperature, salinity, chlorophyll *a *concentration, and sample's water depth. These data offer additional dimensions to analyze the viral PolB fragments identified by our phylogenetic mapping. Here we compared the relative abundance of the predicted viral PolB fragments and the associated metadata across different GOS sampling sites (Figure 4a).

Predicted viral PolB fragments were detected in all of 44 GOS sampling sites (Figure 4b). The relative abundance of different virus groups showed substantial variation across these samples. This is consistent with the diverse ecosystems covered by the GOS expedition.

PolB fragments classified in the phage group were found in 42 (95%) of the 44 sample sites; the two samples without phage PolB fragments were GS08 (Newport Harbor, Richmond, USA) and GS32 (mangrove). In most samples (32 sites), putative phage PolBs exhibited a higher abundance relative to putative eukaryotic viral PolBs. On the other hand, the relative abundance of eukaryotic viral PolBs was higher than that of phage PolBs in 12 sampling sites. We found a significant positive correlation between the relative abundance of phage PolBs and water temperature (*p *= 0.001; Fischer's exact test with no correction for multiple testing): phage-type PolBs showed a higher relative abundance than eukaryotic viral PolBs in tropical waters (T ≥ 20°C), while a reversed tendency was observed in temperate water (T < 20°C). Interestingly, among eukaryotic viral PolBs, putative Mimiviridae PolBs showed the most widespread distribution, being detected in 38 (86%) of the total sites. One of these sampling sites (mangrove located on Isabella, Ecuador) exhibits only viral PolBs classified in the Mimiviridae group. This is the sole mangrove site of all the GOS sampling locations. Mimiviridae PolBs were also relatively abundant in two of the three samples from a hydrostation located in the Sargasso Sea. Three samples correspond to different size fractions: 3.0-20.0 μm for GS01a; 0.8-3.0 μm for GS01b; and 0.1-0.8 μm for GS01c. Putative Mimiviridae PolBs were identified in the GS01a and GS01c samples. The GS01a sample, which was targeted to small eukaryotes, might have contained host species infected by putative viruses of the Mimiviridae group. PolB fragments grouped with chloroviruses were also widely distributed. They were detected in 16 (36%) samples. The relative abundance of this putative eukaryotic virus group showed a significant positive correlation with chlorophyll *a *concentration, a measure of primary productivity in oceanic regions (*p *= 0.00002; Fisher's exact test with no correction for multiple testing).

The sample exhibiting the broadest taxonomic richness of viral PolBs was from Chesapeake Bay (GS12, MD, USA), which is an estuary. The GOS metagenomic sequences from this site exhibited PolB fragments classified in phages, chloroviruses, Asfarviridae and Mimiviridae. Notably, this site is a highly eutrophic estuary with an extremely high chlorophyll *a *concentration. PolBs classified in Asfarviridae were also detected in another estuary site (GS11, Delaware Bay, NY, USA), which is close to Chesapeake Bay.

### Prediction of putative 'new' viral genes

Contigs harboring putative viral PolB homologs were relatively small, ranging from 0.4-12.5 kb (average 1,874 bp) for contigs mapped on eukaryotic viral branches and 0.5-8.8 kb (average 1,885 bp) for phages. To examine the presence of additional open reading frames (ORFs) in these contigs, these putative viral contigs were searched against NRDB using BLASTX. We detected several genes or gene fragments that are usually specific to viruses. For example, several contigs (for example, JCVI SCAF 1096626858151, JCVI SCAF 1096626920680) containing PolB fragments assigned to the chlorovirus group also harbor an ORF most similar to the OtV5 putative major capsid gene. Several putative phage-type contigs (for example, JCVI SCAF 1096628232224, JCVI SCAF 1096626847406) mapped on the cyanophage P-SSM4 branch exhibited ORFs similar to *regA *(translation repressor of early genes) or *uvsX *(*recA*-like recombination and DNA repair protein genes). The presence of such 'virus-specific' genes next to the 'virus-like' PolB homologs corroborates the validity of our phylogenetic mapping approach.

**Figure 5:**
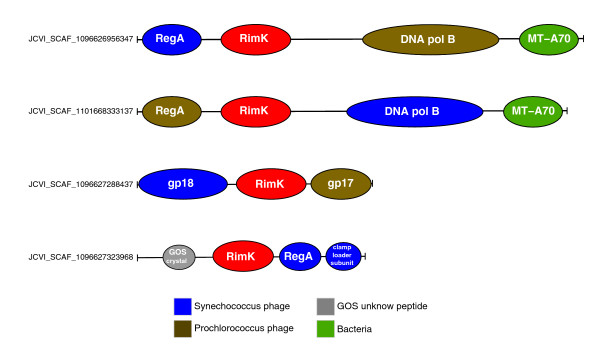
Gene organization of GOS contigs with putative phage RimK sequences. Putative phage *rimK *genes are shown in red. Other predicted genes are color coded according to their best BLAST hit taxonomies in NRDB as shown in the inset panel. MT-A70 corresponds to the adenine-specific methyltransferase. gp17 is a T4-like DNA packaging large subunit terminase homolog. gp18 is a contractile tail sheath protein homolog. The crystal structure of a GOS homolog for the protein encoded by the hypothetical gene (gray) has been determined and is available in the Protein Data Bank (3BY7).

**Figure 6:**
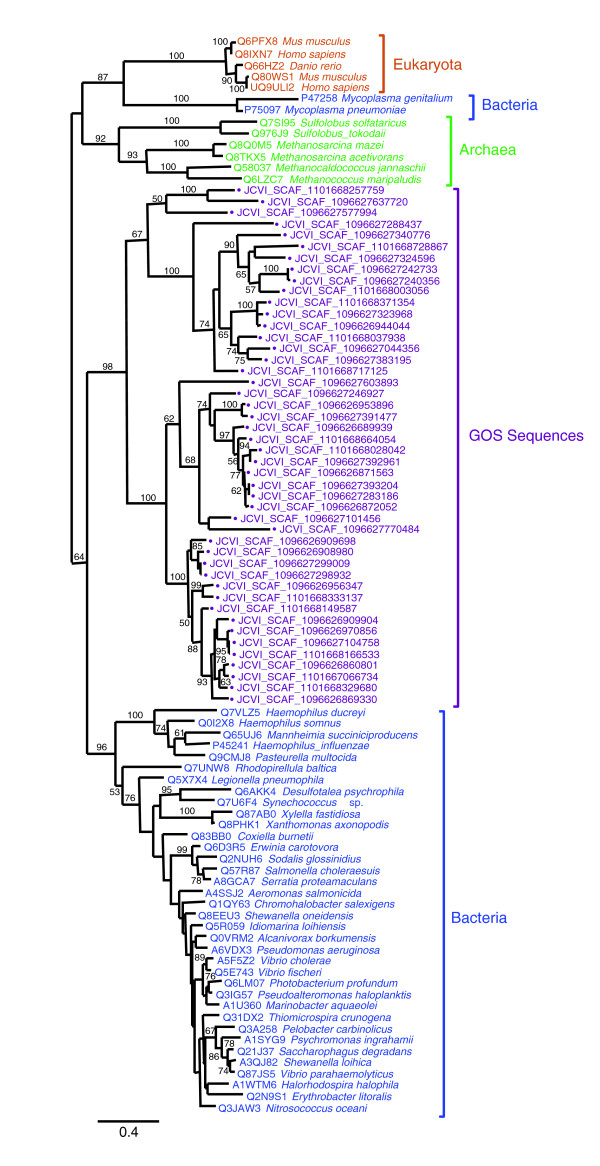
Maximum likelihood tree of RimK sequences. RimK sequences were retrieved from UniProt [78] and from the GOS metagenomic data set using BLASTP. The phylogenetic reconstruction was performed using PhyML [73] (Jones-Taylor-Thornton substitution model [76], 100 bootstrap replicates) based on a multiple sequence alignment generated with MUSCLE [77]. Bootstrap values lower than 50% are not shown. The tree was mid-point rooted. GOS sequences are marked with filled circles and displayed in purple. The length of the scale bar corresponds to 0.4 substitutions per site.

During this search, we found an ORF similar to RimK, a protein involved in post-translational modification of the ribosomal protein S6, in a contig (JCVI SCAF 1096626956347) having a PolB fragment mapped on the cyanophage P-SSM4 branch. In this contig, the *rimK *homolog was flanked by a phage-specific *regA *homolog (Figure 5). *rimK *homologs are found in bacteria, archaea and eukaryotes [41]. To our knowledge, no *rimK *homolog has been found in a viral genome. Using this putative viral RimK homolog as a query of TBLASTN, we screened the entire GOS data set. We identified more than a hundred contigs harboring RimK homologs with higher similarities (BLAST score from 137 up to 732; E-value < 10^-30^) than those exhibited by cellular homologs (BLAST score < 132; E-value > 10^-29^) in NRDB. The sequences of those putative phage RimK homologs were readily aligned with *Escherichia coli *RimK along its entire length (not shown), and showed amino acid residues highly conserved in the ATP-graps domain of bacterial RimK [41]. Several GOS RimK sequences showed an additional domain of unknown function (DUF785, PF05618, E-value < 0.001) at the carboxy-terminal side of the ATP-graps domain. A DUF785 domain is present also in RimK of some bacteria (at the amino-terminal side of the ATP-graps domain) such as *Synechococcus *sp. (Q7U6F4) and euryarchaeotes (at the carboxy-terminal side of the ATP-graps domain) such as *Halobacteria *(for example, Q5V351). Furthermore, many of the GOS contigs encoding RimK homologs exhibited additional ORFs usually specific to phages such as T4-like clamp loader subunit genes, contractile tail sheath protein genes or T4-like DNA packaging large subunit terminase genes (Figure 5). Our phylogenetic analysis indicates that those RimK homologs are closely related to each other and distantly related to bacterial RimK (Figure 6). These results suggest the existence of phages carrying *rimK *homologs in marine environments.

## Discussion

Until recently, the marine virosphere was *terra incognita*. The increasing amount of environmental sequence data now provides unprecedented opportunities to explore the viral world. Previous studies characterized the abundance and the genetic richness of marine viruses using environmental sequencing approaches [8,14,19,23,24]. However, the extent of species diversity within individual viral groups is still unclear. This is especially the case for large DNA viruses. Large DNA viruses were often overlooked or were not the specific focus of marine metagenomic projects. In this study, we used a new phylogenetic mapping approach to identify viral PolB sequences contained in the GOS metagenomic data set and assessed their taxonomic distribution. This study does not concern small viruses, including RNA viruses. Beyond BLAST searches, our phylogenetic mapping approach provided a somewhat unexpected picture of the taxonomic distribution of viral sequences in the metagenomic data.

In the GOS data we identified 811 PolB-like sequences closely related to known viral PolB sequences. This is consistent with the existence of a wide taxonomic spectrum of PolB-containing DNA viruses in marine environments [23]. As previously noted [14], phages are the main contributors to this diversity; our method predicted that 78% (633/811) of the viral PolB fragments were of phage origin. This proportion is likely an underestimate of the actual taxonomic diversity of double-stranded DNA phages in the GOS sampling areas as only a subset of DNA phages carry PolB genes.

Interestingly, the mimivirus group was the second largest in terms of the number of assigned PolB fragments (that is, 115 cases of mapping). Previous studies revealed the existence of mimivirus-like sequences in the GOS metagenomic data set [15,17]. Our data now suggest that the species/strain richness contained in the GOS metagenomic samples for this viral group may be comparable to those exhibited by other groups of eukaryotic large DNA viruses, including most of the previously characterized phycodnaviruses. The amoeba infecting mimivirus has the largest known viral genome (1.2 Mb). Its particle size is approximately 0.7 m in diameter including its filamentous layer [11]. In addition, the mimivirus group contains two haptophyte viruses (CeV01 (510 kb), and PpV01 (485-kb)) and a virus infecting a green algal species (PoV01 (560 kb)) [17,42]. Their genomes are also larger than any other eukaryotic viruses sequenced so far [43,44]. The particle sizes of these three algal viruses are 0.16-0.22 μm, being compatible with the filter sizes used in the GOS sampling. Notably, their particle sizes are comparable to those of classic phycodnaviruses with a mean diameter of 0.16 ± 0.06 μm [45,46]. By counting overlapping PolB fragments mapped on the mimivirus group, we estimated that at least 85 distinct species/strains of Mimiviridae are present in the GOS metagenomic samples. Within the mimivirus group, two haptophyte viruses (PpV1 and CeV01) were clustered together with a high bootstrap value (Figure 3). Most (84%; 97/115) of the Mimiviridae-like PolB fragments were mapped within this subgroup. Haptophyte species may thus be the major hosts of putative viruses corresponding to the PolB subgroup. Overall, these data suggest that large DNA viruses composing the Mimiviridae group represent one of the main components of marine eukaryotic large DNA viruses.

The branch leading to the chloroviruses presented 51 cases of GOS PolB fragment mapping. These GOS sequences were closely related to the recently determined PolB sequence from OtV5. OtV5 infects *Ostreococcus tauri*, a small green algal species of prasinophyte (approximately 1 μm in diameter) found in diverse geographic locations [47]. Short and Suttle identified a group of viral sequences closely related to prasinoviruses (*Micromonas pusilla *viruses) through sequencing PCR products targeted to algal virus PolBs [23]. We found that some of the sequences studied in their work were also highly similar to the OtV5 PolB sequence. For instance, the sequence named BSA99-5 (GenBank: AF405581) in their study exhibited 93% amino acid sequence identity to the OtV5 PolB sequence. This suggests that the major hosts for this putative viral group may be prasinophytes.

Surprisingly, we identified two PolB fragments most closely related to the ASFV PolB. ASFV is currently the sole isolated member of the Asfarviridae family. The known natural hosts of ASFV are terrestrial animals, including warthogs, bush pigs and soft ticks [38]. ASFV causes a persistent but asymptomatic infection in these hosts. In domestic pigs, ASFV causes an acute hemorrhagic infection with mortality rates up to 100% depending on different viral isolates. We now predict the existence of additional Asfarviridae in marine environments, although the contamination from terrestrial origin cannot be excluded. In a recent metagenomic study, Marhaver *et al*. [48] analyzed the viral communities associated with healthy and bleaching corals. They showed that alphaherpesvirus-like and gammaherpesvirus-like sequences accounted for 4-8% of the analyzed environmental sequences. GOS sampling sites include a coral reef atoll site (GS51). No herpesvirus-type PolB fragment was detected in our study.

Through the analysis of geographic distribution, we found that putative viral PolB fragments were identified in all of the 44 GOS samples. This suggests a wide presence of PolB-encoding viruses in diverse marine environments. Interestingly, phage PolB sequences were more abundant than eukaryotic viral PolB sequences in samples from tropical areas; conversely, many samples from temperate areas were enriched in eukaryotic viral PolBs. Further, most of the samples showing a great taxonomic richness of viral PolB sequences corresponded to those from temperate areas. This observation is consistent with the current understanding of the distribution of eukaryotic and bacterial phytoplankton in oceans. Gibb *et al*. [49] surveyed the spatial distributions of phytoplankton pigments across the Atlantic Ocean over 100° of latitude (from 50°N to 50°S). They showed a major transition in pigment characteristics from temperate to tropical/sub-tropical waters; temperate waters were characterized by larger phyto-biomass enriched in eukaryotic phytoplankton, while tropical/sub-tropical waters exhibited smaller phyto-biomass enriched in prokaryotic phytoplankton such as prochlorophytes [49].

The relatively high abundance of eukaryotic viral PolBs in samples from temperate areas (showing high chlorophyll *a *concentrations) was mainly due to the abundance of the GOS PolB sequences grouped with chlorovirus PolBs. This again suggests that the hosts of these putative viruses are green algae (such as prasinophytes). In contrast, Mimiviridae-like PolB fragments showed a wider geographical distribution. They were identified in sequences from most of the GOS sampling sites, from northeast Atlantic Ocean to southwest Pacific Ocean. These sites correspond to a variety of habitat types, such as coastal seas, open oceans, fresh water sites (GS20, Lake Gatun, Panama; GS32, mangrove, Isabella, Ecuador) and even hypersaline waters (GS33, Punta Cormorant Lagoon, Floreana, Ecuador). The detection of Mimiviridae-like PolB fragments was not correlated with chlorophyll *a *concentration. Hence, the hosts of these putative Mimiviridae viruses are not limited in temperate/eutrophic waters. In fact, species of haptophyte have been found and known to occasionally form blooms in waters from sub-polar to (sub-)tropical latitudes, including oligotrophic areas [50-52]. *Acanthamoeba*, the host of mimivirus, also have the ability to survive in diverse environments [53].

Finally, our study allowed the identification of putative phage *rimK*. In *E. coli*, RimK catalyzes the post-translational addition of glutamic acid residues to the amino terminus of ribosomal protein S6 [54]. A resistance to antibiotics was suggested for the *E. coli *mutant lacking the activity of the S6-modification [55]. Reeh and Pedersen [56] showed that the relative level of the S6-modification was not affected by the growth rate in culture. Besides these observations, however, much is unknown for the functional consequence of the S6 modification in *E. coli*. Bacteriophage T7 modifies ribosomal protein S6, S1 and translational initiation factors by phosphorylation upon infection of *E. coli *[57]. The modifications of host translational proteins are performed by a T7-encoded kinase, and enhance phage reproduction under sub-optimal growth conditions. It was suggested that the phosphorylation of these proteins serves to stimulate translation of the phage late mRNAs. The RimK homologs found in phage-like contigs may be involved in a similar process. Unexpected homologs of cellular genes are continuously identified in viral genome sequences [12,58,59]. We believe that our phylogenetic mapping approach will be useful to identify further occurrences of unexpected viral genes in environmental sequences.

## Conclusion

The use of a phylogenetic approach provided a comprehensive picture of the taxonomic distribution of large viruses enclosed in the GOS metagenomic data. As expected, the highest genetic richness corresponded to phages. Interestingly, our data suggest that Mimiviridae represent a major and ubiquitous component of large eukaryotic DNA viruses in diverse marine environments.

## Materials and methods

### Extraction of PolB fragments from the GOS metagenomic data set

We retrieved the combined assemblies of the GOS metagenomic data through the CAMERA website [60]. The data set was composed of 3,081,849 scaffolds. We extracted all the stop-to-stop ORFs (≥ 60 amino acid residues) from the assembled sequences using EMBOSS/GETORF [61]. We obtained a set of 21,406,171 ORFs. Those ORFs were translated into corresponding amino acid sequences. To identify PolB-like fragments in this set, we used the Pfam profile (PF00136, both long and fragment search versions: 'ls' and 'fs') [62] and the HMMER software as a search engine [63] using an E-value threshold of 0.001. We then removed redundancy (due to the double use of 'ls' and 'fs' versions of the Pfam profile) and false positive detections (having the best hit against non-PolB sequences in the NRDB) by BLASTP [64] using an E-value threshold of 10^-5^). We extracted only the parts of metagenomic amino acid sequences that were aligned on the Pfam profile representing the polymerase domains of PolB. Thus, additional domains (such as endonuclease domains) were not included in our PolB sequence set. No contig was found to contain more than one PolB homolog. As a result of these processes, we obtained 1,947 distinct PolB-like sequences (from 23-562 amino acid residues); these sequences are referred to as PolB fragments in this study. We parsed the GOS PolB fragments to find intein insertions by the TIGRFAM profiles TIGR01445 (intein amino terminus) and TIGR01443 (intein carboxyl terminus) [65], but none of these fragments had a detectable intein domain. In this study, we did not include the protein priming subfamily of the B family DNA polymerase [28], which is represented by the Pfam profile PF03175. The members of this subfamily are found in eukaryotic linear plasmids of mitochondrion, phages and adenoviruses.

### PolB homologs from the NRDB

We retrieved PolB homologs from the NRDB, RefSeq [66] and KEGG [67] databases using BLAST using multiple query sequences (E-value < 10^-5^) and the PolB Pfam profile (E-value < 0.001). We removed species redundancy using BLASTCLUST [64] while keeping the widest possible taxonomic/paralog coverage (but with a non-exhaustive sampling for closely related species). This resulted in a set of 120 PolB homologs (Additional data file 1). We removed intein sequences in the PolBs of mimivirus [68], HaV [69] and CeV01 (GenBank: ABU23716).

### Construction of the reference alignment and the reference tree

We next constructed an alignment of PolB homologs from known organisms (that is, the reference MSA) and generated a phylogenetic tree of PolB homologs (that is, the reference tree). There is a tradeoff between the number of distant homologs included in the reference MSA (contributing to a wider taxonomic/paralog coverage) and the quality of the resulting MSA and tree (contributing to a reliable classification of metagenomic sequences). Among the 120 PolB homologs, we identified 19 highly divergent sequences that decrease the quality of the resulting PolB alignment and tree but that show no close homologs in the GOS PolB fragments. This process was performed through multiple trials of building alignments by T-Coffee [70] and phylogenetic trees by PhyML for the PolB homologs. These 19 sequences correspond to six groups of PolB homologs: eukaryotic DNA polymerase ε, a *Trichomonas vaginalis *DNA polymerase α-like paralog, PolBs of unclassified herpesviruses (Ostreid, Ictalurid and Ranid herpesviruses), *Heliothis zea *virus, a nimavirus (shrimp white spot syndrome virus), and PolBs of a group of bacteria related to *Prosthecochloris vibrioformis *and *Chlorobium tepidum*. There was no PolB-like fragment in the GOS data exhibiting a best BLAST hit against these groups of PolB homologs. Therefore, the removal of the six groups of PolB homologs from our reference data set does not affect the interpretation of the results described in this manuscript. After discarding these 19 sequences, the final PolB set was composed of 101 sequences. We aligned the 101 PolB sequences using M-Coffee accessible from a public server [71] with the use of default options. M-Coffee is a meta-method for assembling multiple sequence alignments [72]. We extracted only the polymerase domain sequences from the alignment (that is, the reference MSA; Additional data file 2). The reference alignment showed four conserved regions (numbered from I to IV) previously described as the signatures of the PolB polymerase domains [33]. We next built a maximum likelihood tree based on the reference MSA (that is, the reference tree) using PhyML after removing gap-containing sites [73] with JTT substitution model and a gamma low (four rate categories). Bootstrap values were obtained after 100 bootstrap replicates. We used the phylogeny.fr platform [74] to generated scalable vector graphics from newick formatted trees.

### Phylogenetic mapping

Each of the metagenomic PolB fragments was taxonomically assigned by aligning it against the reference MSA and by examining its phylogenetic position in the reference tree. In order to reduce the computation time and to avoid unnecessary complications in summarizing results within too dense a tree, we reduced the size of the reference MSA and the reference tree. Specifically, we selected 51 PolBs from the 101 PolBs contained in the initial set. We kept the selected 51 PolBs in the reduced set, and deleted the remaining PolBs. The selection of the 51 representatives was carried out in the following way. First, we selected all the PolBs (that is, ASFV, EhV86, HaV, Phage RM378) that were not grouped with other PolBs with a statistical support (≥ 70% bootstrap value) in the initial reference tree (Figure 1). Second, we selected two or three representatives from each of the statistically supported monophyletic groups (≥ 70% bootstrap value). The choice of representatives from a monophyletic group was arbitrary. We simply selected two or three relatively distant sequences from the members of the monophyletic group. To obtain a reduced reference MSA composed of the selected 51 sequences, we extracted a part (that is, lines) of the initial reference MSA (containing gaps). The initial reference tree (composed of 199 branches including internal ones) was also reduced by pruning branches leading to the non-selected leaves using BAOBAB [75].

The reduced reference tree has 99 branches (including internal branches); the constraint on the topology of the reduced reference tree thus defined 99 possible branching positions for each PolB-like fragment extracted from the metagenomic data set. The reduced reference MSA and the reduced reference tree are the basis for our phylogenetic mapping in this study. Each of the PolB fragments from the GOS data set was aligned on the reduced reference MSA (containing gaps) using T-Coffee [70] with a profile alignment option. For the T-Coffee profile alignment, we used the option '-profile comparison = full10'. If a GOS PolB fragment generates an alignment with less than 50 sites after removing gap-containing sites, we discarded the GOS PolB fragment from our analysis. Based on the resulting alignment (51 reference sequences and one GOS PolB fragment), the likelihoods of all 99 possible branching positions (thus 99 different topologies) for the PolB fragment were computed by ProtML [35]. A statistical significance for the best tree among the 99 topologies was assessed by the RELL method [36,37]. We considered the branching position of a PolB fragment to be supported when the RELL bootstrap value for the best topology was ≥ 75%.

### Read coverage

Read coverage for a contig was defined by dividing the cumulated size of reads contributing to the contig by the size of the contig.

### Relative abundance of PolBs

For the analysis of the relative abundance of PolB sequences, we used the same approach used by Williamson *et al*. [14]. Briefly, we first estimated the average number of reads overlapping with a part of a contig where a PolB domain was encoded, by taking into account the length of the PolB domain (as defined by the Pfam hit) and the length of the contig. The abundance of the PolB-sequences for each viral group for a given sample site was then quantified by the total number of reads associated with the relevant set of PolB-sequences (that is, the sum of the estimated read numbers). For a given site, the viral PolB proportion was computed by dividing the total number of viral PolB reads (for all viral groups) by the total number of reads obtained from the site.

## Abbreviations

ASFV, African swine fever virus; CeV, *Chrysochromulina ericina *virus; EhV86, *Emiliania huxleyi *virus 86; GOS, Global Ocean Sampling; HaV, *Heterosigma akashiwo *virus 1; MSA, multiple sequence alignment; NCLDV, nucleocytoplasmic large DNA virus; NRDB, NCBI non-redundant amino-acid sequence database; ORF, open reading frame; PolB, B-family DNA polymerase; PoV, *Pyramimonas orientalis *virus; PpV, *Phaeocystis pouchetii *virus; RELL, resampling of estimated log likelihoods.

## Authors' contributions

AM performed the analyses. HO designed the experiments. All authors analyzed the data and contributed to the writing of the manuscript.

## Additional data files

The following additional data are available with the online version of this paper. Additional data file 1 is a table listing the PolB sequences used in the study. Additional data file 2 is a multiple sequence alignment of 101 PolB sequences retrieved from databases. Additional data file 3 is a figure summarizing the results of the phylogenetic mapping of the GOS PolB fragments, which are displayed for each of the 99 branches tested.

## Supplementary Material

Additional data file 1 The IDs and species names of the PolB sequences retrieved from databases are given. Sequences used in the reference multiple alignment are in bold. Click here for file

Additional data file 2 Sequences used in the final reduced reference multiple alignment are displayed with an asterisk. Click here for file

Additional data file 3 The GOS PolB fragments are displayed for each of the 99 branches tested. Numbers in parentheses (*V/W*) are the total number of mapped PolB fragments (*W*) and the number of supported cases (*V*) (displayed in red). Read coverage values are presented as follows: [*X*-*Y*]-(*Z*) where X and Y are the read coverage value range (minimum/maximum) and *Z *the read coverage median value. Click here for file
