# Supplementary material for: Taxonomic distribution of large DNA viruses in the sea
Source: Genome Biol. 2008 Jul 3;9(7):R106. doi: 10.1186/gb-2008-9-7-r106 (PMC2530865; doi:10.1186/gb-2008-9-7-r106)
Supplement: Additional data file 1 — The IDs and species names of the PolB sequences retrieved from databases are given. Sequences used in the reference multiple alignment are in bold. [file gb-2008-9-7-r106-S1.pdf]

List of the PolB sequences used in this study. IDs are displayed as retrieved in NRDB, RefSeq and KEGG databases. Selected sequences used in the reference multiple sequence alignment are displayed in bold.

IDs	Species	Annotation
**AAA58439**	***Homo sapiens***	**DNA polymerase-delta catalytic-subunit**
**AAC62689**	***Cenarchaeum symbiosum***	**archael family B DNA polymerase**
**AAG09402**	***Homo sapiens***	**DNA polymerase zeta catalytic subunit variant 1**
AAK14825	*Plasmodium falciparum*	DNA polymerase alpha
**AAR26842**	***Feldmannia irregularis* virus a**	**FirrV-1-A18**
AAZ32459	uncultured euryarchaeote Alv-FOS1	DNA polymerase family B
**ABU2317**	***Pyramimonas orientalis* virus**	**putative B family DNA polymerase I**
ABU2318	*Phaeocystis pouchetii* virus	putative B family DNA polymerase I
**ABU23716**	***Chrysochromulina ericina* virus**	**putative B family DNA polymerase I**
BAA35142	*Paramecium bursaria* Chlorella virus CVK2	DNA polymerase
**BAE06251**	***Heterosigma akashiwo* virus 01**	**B-family DNA polymerase**
**BAE19749**	**Human herpesvirus 1**	**DNA polymerase UL30**
**CAC84471**	***Heliothis virescens* ascovirus 3c**	**delta DNA polymerase**
CMI176C *	*Cyanidioschyzon merolae*	DNA polymerase alpha, subunit A
CMQ098C * #	*Cyanidioschyzon merolae*	DNA polymerase epsilon, subunit A
CMR103C *	*Cyanidioschyzon merolae*	DNA polymerase zeta subunit
NP_006222 #	*Homo sapiens*	DNA polymerase epsilon catalytic subunit
**NP_039988**	**Human herpesvirus 5 strain AD169**	**UL54**
NP_041148 #	Ictalurid herpesvirus 1	ORF57
NP_042094	Variola virus	hypothetical protein VARVgp050
**NP_042783**	**African swine fever virus**	**DNA polymerase**
NP_043990	Molluscum contagiosum virus	MC039L
**NP_048107**	***Melanoplus sanguinipes* entomopoxvirus**	**ORF MSV036 putative DNA polymerase**
**NP_048532**	***Paramecium bursaria* Chlorella virus 1**	**hypothetical protein PBCV1_A185R**

**NP_049662**	**Enterobacteria phage T4**	**gp43 DNA polymerase**
NP_051748	Myxoma virus	m34L
**NP_058633**	***Homo sapiens***	**DNA polymerase alpha catalytic subunit**
NP_064832	*Amsacta moorei* entomopoxvirus 'L'	DNA-directed DNA polymerase
**NP_069333**	***Archaeoglobus fulgidus* DSM 4304**	**DNA polymerase B1 (polB)**
NP_073424	Yaba-like disease virus	39L protein
**NP_077578**	***Ectocarpus siliculosus* virus 1**	**EsV-1-93**
NP_146963	*Aeropyrum pernix* K1	family B DNA polymerase I
NP_148383	*Aeropyrum pernix* K1	family B DNA polymerase II
**NP_148473**	***Aeropyrum pernix* K1**	**DNA polymerase II**
**NP_148895**	***Cydia pomonella* granulovirus**	**ORF111 DNAPOL**
**NP_149500**	**Invertebrate iridescent virus 6**	**037L**
**NP_203396**	***Culex nigripalpus* NPV**	**CUN091 putative dnapolymerase, similar to AcMNPV ORF65**
**NP_279569**	**Halobacterium sp. NRC-1**	**DNA polymerase B1**
NP_342079	*Sulfolobus solfataricus* P2	DNA polymerase II
**NP_342896**	***Sulfolobus solfataricus* P2**	**DNA polymerase II (DNA polymerase B2) carboxy-end (dpo2)**
**NP_378066**	***Sulfolobus tokodaii* str. 7**	**DNA polymerase II**
NP_393928	*Thermoplasma acidophilum* DSM 1728	DNA polymerase II
**NP_394366**	***Thermoplasma acidophilum* DSM 1728**	**DNA polymerase II**
NP_478036 #	Shrimp white spot syndrome virus	wsv514
**NP_542554**	**Halorubrum phage HF2**	**putative DNA-dependent DNA polymerase**
**NP_559083**	***Pyrobaculum aerophilum* str. IM2**	**possible DNA-directed DNA polymerase (B2)**
**NP_559770**	***Pyrobaculum aerophilum* str. IM2**	**DNA polymerase II**
**NP_559825**	***Pyrobaculum aerophilum* str. IM2**	**DNA polymerase II**
NP_570196	Swinepox virus	SPV036 DNA polymerase
NP_577941	*Pyrococcus furiosus* DSM 3638	DNA-directed DNA polymerase
NP_586236 #	*Encephalitozoon cuniculi* GB-M1	DNA polymerase epsilon

NP_597442	*Encephalitozoon cuniculi* GB-M1	DNA-directed DNA polymerase alpha
NP_612241	Infectious spleen and kidney necrosis virus	putative DNA polymerase
**NP_614322**	***Methanopyrus kandleri* AV19**	**B family DNA polymerase**
**NP_615844**	***Methanosarcina acetivorans* C2A**	**DNA-directed DNA polymerase**
NP_663155 #	*Chlorobium tepidum* TLS	DNA polymerase family B protein
NP_690550 #	*Heliothis zea* virus 1	DNA polymerase I
**NP_835679**	**Rhodothermus phage RM378**	**similar to DNA-directed DNA polymerase**
NP_899330	Vibrio phage KVP40	DNA polymerase
NP_943895	Aeromonas phage Aeh1	DNA polymerase
NP_954286 #	*Geobacter sulfurreducens* PCA	DNA polymerase II, putative
**NP_955144**	**Canarypox virus**	**CNPV121 DNA polymerase**
**P30320**	***Paramecium bursaria* Chlorella virus NY2A**	**DNA polymerase**
**P56689**	***Thermococcus gorgonarius***	**DNA polymerase (TO POL)**
XP_001011832	*Tetrahymena thermophila* SB210	DNA polymerase family B containing protein
XP_001013747	*Tetrahymena thermophila* SB210	DNA polymerase catalytic subunit
XP_001017761	*Tetrahymena thermophila* SB210	DNA polymerase family B containing protein
XP_001032353	*Tetrahymena thermophila* SB210	DNA polymerase family B containing protein
XP_001301333 #	*Trichomonas vaginalis* G3	hypothetical protein TVAG_050650
XP_001303643	*Trichomonas vaginalis* G3	DNA polymerase family B containing protein
**XP_001306852**	***Trichomonas vaginalis* G3**	**polymerase alpha subunit, putative**
**XP_001326973**	***Trichomonas vaginalis* G3**	**polymerase zeta subunit, putative**
XP_001347646	*Plasmodium falciparum* 3D7	DNA polymerase zeta catalytic subunit, putative
**XP_001683479**	***Leishmania major* strain Friedlin**	**DNA polymerase zeta catalytic subunit, putative**
XP_001685930	*Leishmania major* strain Friedlin	DNA polymerase delta catalytic subunit, putative
XP_001707891	*Giardia lambia*	DNA polymerase delta, catalytic subunit
XP_626972	*Cryptosporidium parvum* Iowa II	DNA polymerase alpha catalytic subunit

XP_627031 #	*Cryptosporidium parvum* Iowa II	DNA polymerase epsilon catalytic subunit
XP_638283	*Dictyostelium discoideum* AX4	DNA polymerase delta catalytic subunit
XP_639211 #	*Dictyostelium discoideum* AX4	putative DNA polymerase epsilon subunit A
XP_640277	*Dictyostelium discoideum* AX4	DNA polymerase alpha catalytic subunit
XP_645553	*Dictyostelium discoideum* AX4	DNA polymerase zeta catalytic subunit
XP_654477	*Entamoeba histolytica* HM-1	DNA polymerase delta catalytic subunit
XP_656768	*Entamoeba histolytica* HM-1	DNA polymerase zeta catalytic subunit
XP_657373	*Entamoeba histolytica* HM-1	DNA polymerase alpha catalytic subunit
XP_763220	*Theileria parva* strain Muguga	DNA polymerase alpha
XP_843543 #	*Leishmania major* strain Friedlin	DNA polymerase epsilon catalytic subunit, putative
XP_847160	*Trypanosoma brucei* TREU927	DNA polymerase zeta catalytic subunit, putative
XP_847318	*Trypanosoma brucei* TREU927	DNA polymerase alpha catalytic subunit
XP_951513	*Trypanosoma brucei* TREU927	DNA polymerase delta catalytic subunit
XP_955596	*Encephalitozoon cuniculi* GB-M1	DNA polymerase delta catalytic subunit
XP_966285 #	*Plasmodium falciparum* 3D7	DNA polymerase epsilon, catalytic subunit a, putative
**YP_001055588**	***Pyrobaculum calidifontis* JCM 11548**	**DNA polymerase B region**
**YP_001097770**	***Methanococcus maripaludis* C5**	**DNA polymerase Pol2**
YP_001129537 #	*Prosthecochloris vibrioformis* DSM 265	DNA polymerase B region
YP_003817	*Ambystoma tigrinum* virus	DNA polymerase
YP_024639 #	*Ostreid herpesvirus* 1	ORF100
**YP_025135**	***Neodiprion sertifer* NPV**	**DNA polymerase**
**YP_073706**	**Lymphocystis disease virus - isolate China**	**DNA polymerase elongation subunit family B**
YP_136425	Haloarcula marismortui ATCC 43049	DNA polymerase B elongation subunit
**YP_142676**	***Acanthamoeba polyphaga* mimivirus**	**DNA polymerase (B family)**
YP_195168	Cyanophage phage S-PM2	DNA polymerase gp43
YP_214414	Cyanophage P-SSM2	T4-like DNA polymerase
**YP_214707**	**Cyanophage P-SSM4**	**gp43**

**YP_293784**	***Emiliania huxleyi* virus 86**	**putative DNA polymerase delta catalytic subunit**
YP_320359 #	*Anabaena variabilis*	DNA polymerase I-like
YP_401712	Human herpesvirus 4 type 1	BALF5
YP_502623	*Methanospirillum hungatei* JF-1	DNA polymerase B region
YP_656618 #	Ranid herpesvirus 2	DNA polymerase subunit catalytic subunit
YP_656727 #	Ranid herpesvirus 1	ORF72
**YP_684489**	**uncultured methanogenic archaeon RC-I**	**DNA-directed DNA polymerase B**
**YP_687101**	**uncultured methanogenic archaeon RC-I**	**putative DNA-directed DNA polymerase B**
YP_717843	Phage Syn9	gp176
**YP_751308**	***Shewanella frigidimarina* NCIMB 400**	**DNA polymerase B region**
YP_762356	*Spodoptera frugiperda* ascovirus 1a	125.8 kDa Delta DNA Polymerase
**YP_803224**	***Trichoplusia ni* ascovirus 2c**	**DNA polymerase**
**YP_843812**	***Methanosaeta thermophila* PT**	**DNA polymerase Pol2**
YP_856637	*Aeromonas hydrophila* subsp. hydrophila ATCC 7966	DNA polymerase II
**ZP_00923866**	***Escherichia coli* 101-1**	**COG0417: DNA polymerase elongation subunit (family B)**

(*) KEGG ID; (#) Sequences not included in the complete reference set.
