# Supplementary material for: Taxonomic distribution of large DNA viruses in the sea
Source: Genome Biol. 2008 Jul 3;9(7):R106. doi: 10.1186/gb-2008-9-7-r106 (PMC2530865; doi:10.1186/gb-2008-9-7-r106)

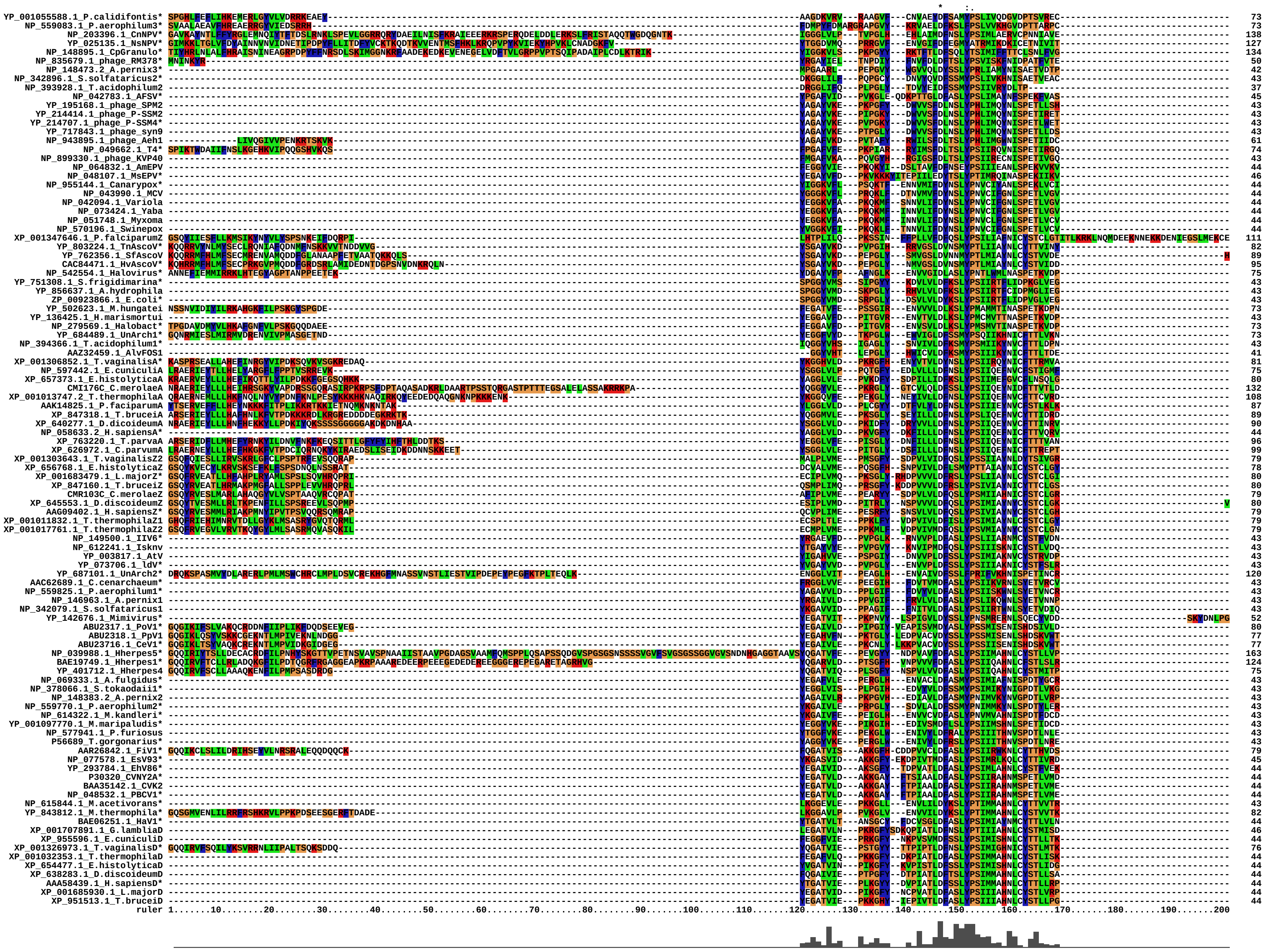

YP_001055588.1_P.calidifontis* 73
NP_559083.1_P.aerophilum3* 73
NP_203396.1_CnNPV* 138
YP_025135.1_NsNPV* 127
NP_148895.1_CpGranulo* 134
NP_835679.1_phage_RM378* 50
NP_148473.2_A.pernix3* 42
NP_342896.1_S.solfataricus2* 43
NP_393928.1_T.acidophilum2 37
NP_042783.1_AFSV* 45
YP_195168.1_phage_SPM2 43
YP_214414.1_phage_P-SSM2 43
YP_214707.1_phage_P-SSM4* 43
YP_717843.1_phage_syn9 43
NP_943895.1_phage_Aeh1 61
NP_049662.1_T4* 74
NP_899330.1_phage_KVP40 43
NP_064832.1_AmEPV 44
NP_048107.1_MsEPV* 46
NP_955144.1_Canarypox* 44
NP_043990.1_MCV 44
NP_042094.1_Variola 44
NP_073424.1_Yaba 44
NP_051748.1_Myxoma 44
NP_570196.1_Swinepox 44
XP_001347646.1_P.falciparumZ 111
YP_803224.1_TnAscoV* 82
YP_762356.1_SfAscoV 89
CAC84471.1_HvAscoV* 95
NP_542554.1_Halovirus* 75
YP_751308.1_S.frigidimarina* 43
YP_856637.1_A.hydrophila 43
ZP_00923866.1_E.coli* 43
YP_502623.1_M.hungatei 73
YP_136425.1_H.marismortui 43
NP_279569.1_Halobact* 73
YP_684489.1_UnArch1* 73
NP_394366.1_T.acidophilum1* 43
AAZ32459.1_AlvFOS1 41
XP_001306852.1_T.vaginalisA* 81
NP_597442.1_E.cuniculiA 75
XP_657373.1_E.histolyticaA 80
CMI176C_C.merolaeA 132
XP_001013747.2_T.thermophilaA 108
AAK14825.1_P.faciparumA 87
XP_847318.1_T.bruceiA 89
XP_640277.1_D.dicoideumA 90
NP_058633.2_H.sapiensA* 44
XP_763220.1_T.parvaA 96
XP_626972.1_C.parvumA 99
XP_001303643.1_T.vaginalisZ2 79
XP_656768.1_E.histolyticaZ 78
XP_001683479.1_L.majorZ* 80
XP_847160.1_T.bruceiZ 80
CMR103C_C.merolaeZ 79
XP_645553.1_D.discoideumZ 80
AAG09402.1_H.sapiensZ* 79
XP_001011832.1_T.thermophilaZ1 79
XP_001017761.1_T.thermophilaZ2 79
NP_149500.1_IIV6* 43
NP_612241.1_Isknv 43
YP_003817.1_AtV 43
YP_073706.1_ldV* 43
YP_687101.1_UnArch2* 120
AAC62689.1_C.cenarchaeum* 43
NP_559825.1_P.aerophilum1* 43
NP_146963.1_A.pernix1 43
NP_342079.1_S.solfataricus1 43
YP_142676.1_Mimivirus* 52
ABU2317.1_PoV1* 80
ABU2318.1_PpV1 77
ABU23716.1_CeV1* 77
NP_039988.1_Hherpes5* 163
BAE19749.1_Hherpes1* 124
YP_401712.1_Hherpes4 75
NP_069333.1_A.fulgidus* 43
NP_378066.1_S.tokadaii1* 43
NP_148383.2_A.pernix2 43
NP_559770.1_P.aerophilum2* 43
NP_614322.1_M.kandleri* 43
YP_001097770.1_M.maripaludis* 43
NP_577941.1_P.furiosus 43
P56689_T.gorgonarius* 43
AAR26842.1_FiV1* 79
NP_077578.1_EsV93* 45
YP_293784.1_EhV86* 44
P30320_CVNY2A* 44
BAA35142.1_CVK2 44
NP_048532.1_PBCV1* 44
NP_615844.1_M.acetivorans* 43
YP_843812.1_M.thermophila* 82
BAE06251.1_HaV1* 44
XP_001707891.1_G.lambliaD 46
XP_955596.1_E.cuniculiD 44
XP_001326973.1_T.vaginalisD* 76
XP_001032353.1_T.thermophilaD 44
XP_654477.1_E.histolyticaD 44
XP_638283.1_D.discoideumD 44
AAA58439.1_H.sapiensD* 44
XP_001685930.1_L.majorD 44
XP_951513.1_T.bruceiD 44
ruler 1.......10........20........30........40........50........60........70........80........90.......100.......110.......120.......130.......140.......150.......160.......170.......180.......190.......200

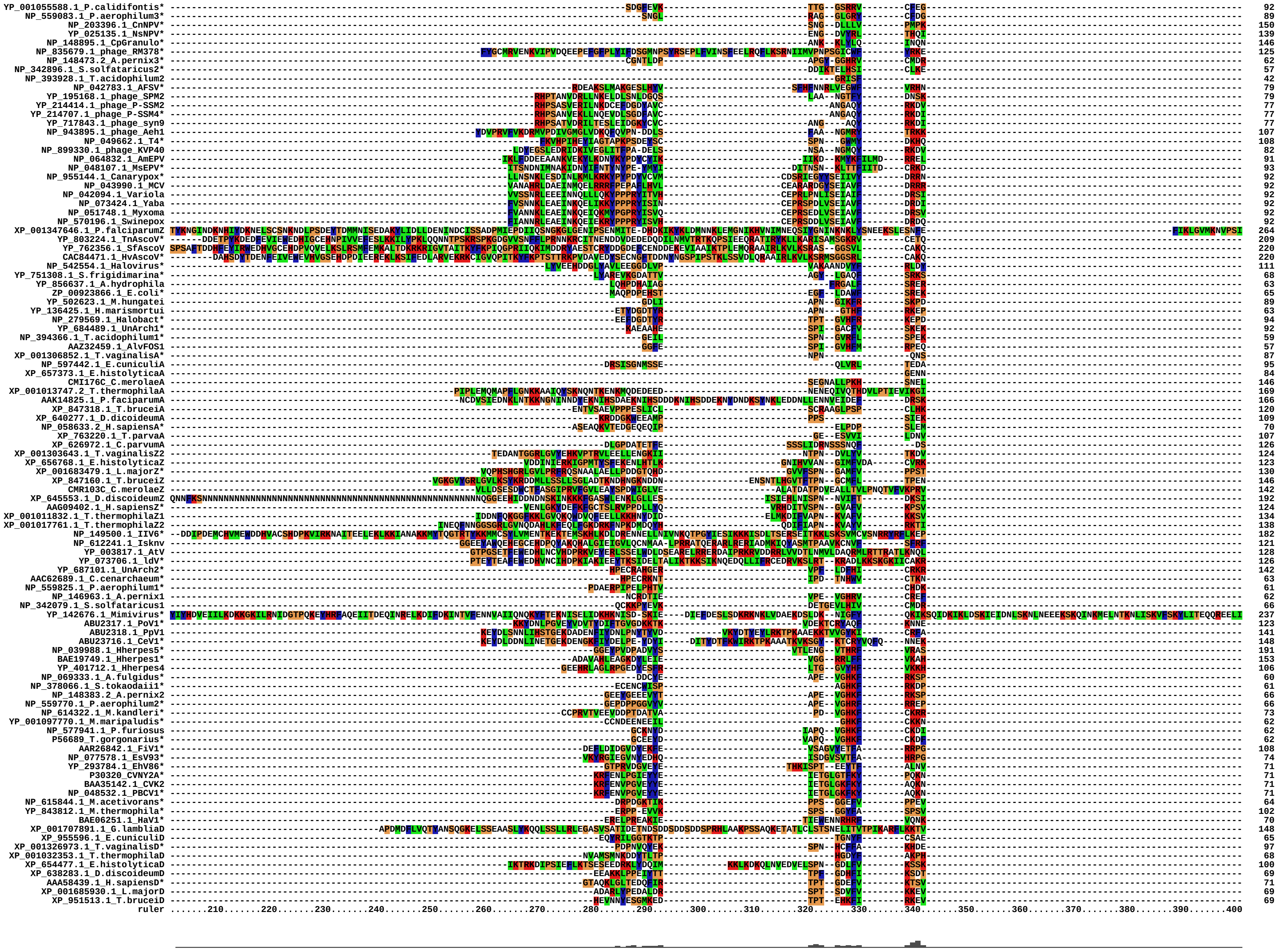
YP_001055588.1_P.calidifontis* 92
NP_559083.1_P.aerophilum3* 89
NP_203396.1_CnNPV* 150
YP_025135.1_NsNPV* 139
NP_148895.1_CpGranulo* 146
NP_835679.1_phage_RM378* 125
NP_148473.2_A.pernix3* 62
NP_342896.1_S.solfataricus2* 57
NP_393928.1_T.acidophilum2 42
NP_042783.1_AFSV* 79
YP_195168.1_phage_SPM2 79
YP_214414.1_phage_P-SSM2 77
YP_214707.1_phage_P-SSM4* 77
YP_717843.1_phage_syn9 77
NP_943895.1_phage_Aeh1 107
NP_049662.1_T4* 108
NP_899330.1_phage_KVP40 82
NP_064832.1_AmEPV 91
NP_048107.1_MsEPV* 93
NP_955144.1_Canarypox* 92
NP_043990.1_MCV 92
NP_042094.1_Variola 92
NP_073424.1_Yaba 92
NP_051748.1_Myxoma 92
NP_570196.1_Swinepox 92
XP_001347646.1_P.falciparumZ 264
YP_803224.1_TnAscoV* 209
YP_762356.1_SfAscoV 220
CAC84471.1_HvAscoV* 220
NP_542554.1_Halovirus* 111
YP_751308.1_S.frigidimarina* 68
YP_856637.1_A.hydrophila 63
ZP_00923866.1_E.coli* 65
YP_502623.1_M.hungatei 89
YP_136425.1_H.marismortui 63
NP_279569.1_Halobact* 94
YP_684489.1_UnArch1* 92
NP_394366.1_T.acidophilum1* 59
AAZ32459.1_AlvFOS1 57
XP_001306852.1_T.vaginalisA* 87
NP_597442.1_E.cuniculiA 95
XP_657373.1_E.histolyticaA 84
CMI176C_C.merolaeA 146
XP_001013747.2_T.thermophilaA 169
AAK14825.1_P.faciparumA 166
XP_847318.1_T.bruceiA 120
XP_640277.1_D.dicoideumA 109
NP_058633.2_H.sapiensA* 70
XP_763220.1_T.parvaA 107
XP_626972.1_C.parvumA 126
XP_001303643.1_T.vaginalisZ2 124
XP_656768.1_E.histolyticaZ 123
XP_001683479.1_L.majorZ* 130
XP_847160.1_T.bruceiZ 146
CMR103C_C.merolaeZ 142
XP_645553.1_D.discoideumZ 192
AAG09402.1_H.sapiensZ* 124
XP_001011832.1_T.thermophilaZ1 134
XP_001017761.1_T.thermophilaZ2 138
NP_149500.1_IIV6* 182
NP_612241.1_Isknv 121
YP_003817.1_AtV 128
YP_073706.1_ldV* 126
YP_687101.1_UnArch2* 142
AAC62689.1_C.cenarchaeum* 63
NP_559825.1_P.aerophilum1* 61
NP_146963.1_A.pernix1 62
NP_342079.1_S.solfataricus1 66
YP_142676.1_Mimivirus* 237
ABU2317.1_PoV1* 123
ABU2318.1_PpV1 141
ABU23716.1_CeV1* 148
NP_039988.1_Hherpes5* 191
BAE19749.1_Hherpes1* 153
YP_401712.1_Hherpes4 106
NP_069333.1_A.fulgidus* 60
NP_378066.1_S.tokadaii1* 61
NP_148383.2_A.pernix2 66
NP_559770.1_P.aerophilum2* 66
NP_614322.1_M.kandleri* 73
YP_001097770.1_M.maripaludis* 62
NP_577941.1_P.furiosus 62
P56689_T.gorgonarius* 62
AAR26842.1_FiV1* 108
NP_077578.1_EsV93* 74
YP_293784.1_EhV86* 71
P30320_CVNY2A* 71
BAA35142.1_CVK2 71
NP_048532.1_PBCV1* 71
NP_615844.1_M.acetivorans* 64
YP_843812.1_M.thermophila* 102
BAE06251.1_HaV1* 70
XP_001707891.1_G.lambliaD 148
XP_955596.1_E.cuniculiD 65
XP_001326973.1_T.vaginalisD* 97
XP_001032353.1_T.thermophilaD 68
XP_654477.1_E.histolyticaD 100
XP_638283.1_D.discoideumD 69
AAA58439.1_H.sapiensD* 71
XP_001685930.1_L.majorD 69
XP_951513.1_T.bruceiD 69
ruler 210 220 230 240 250 260 270 280 290 300 310 320 330 340 350 360 370 380 390 400

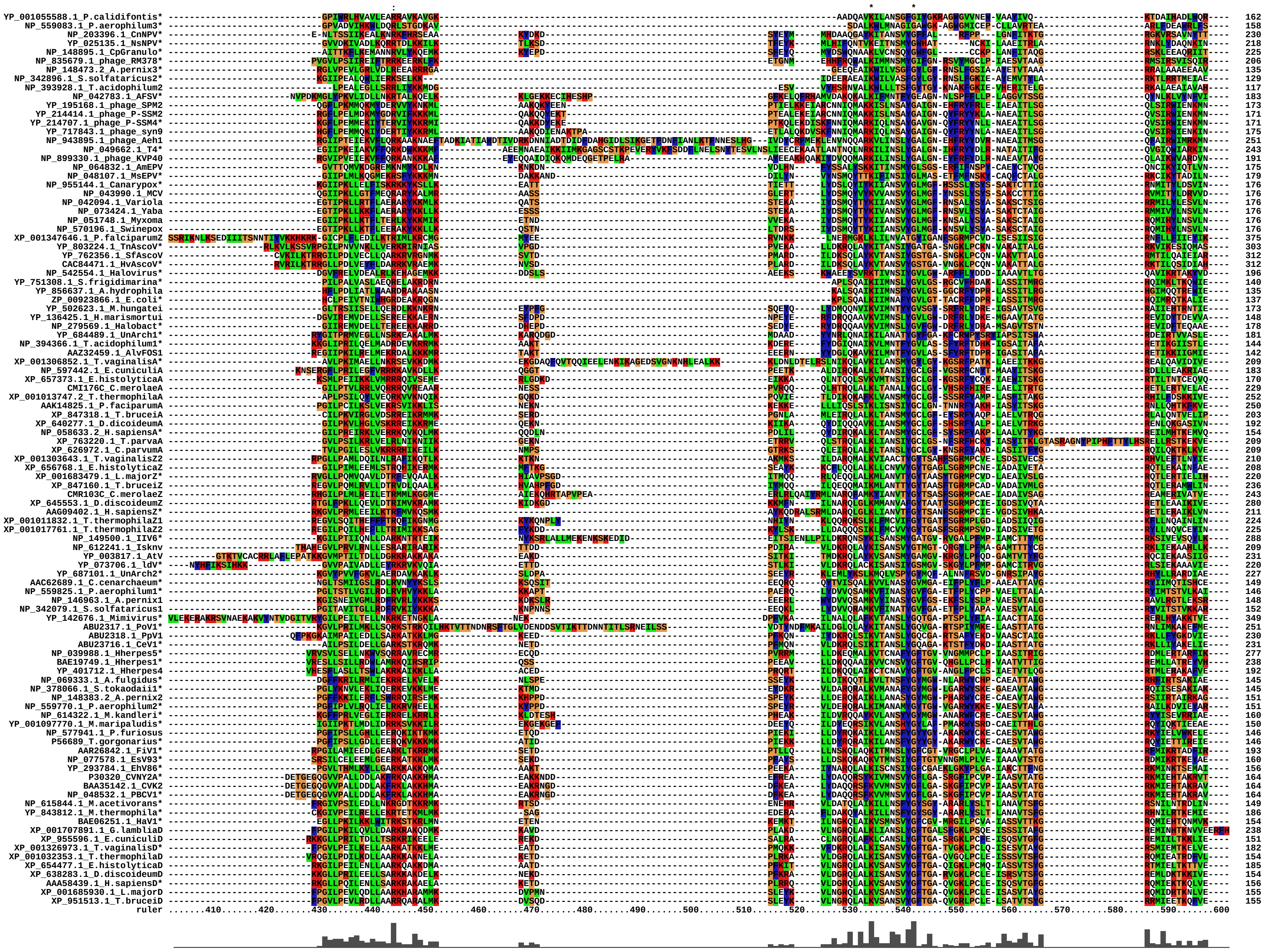

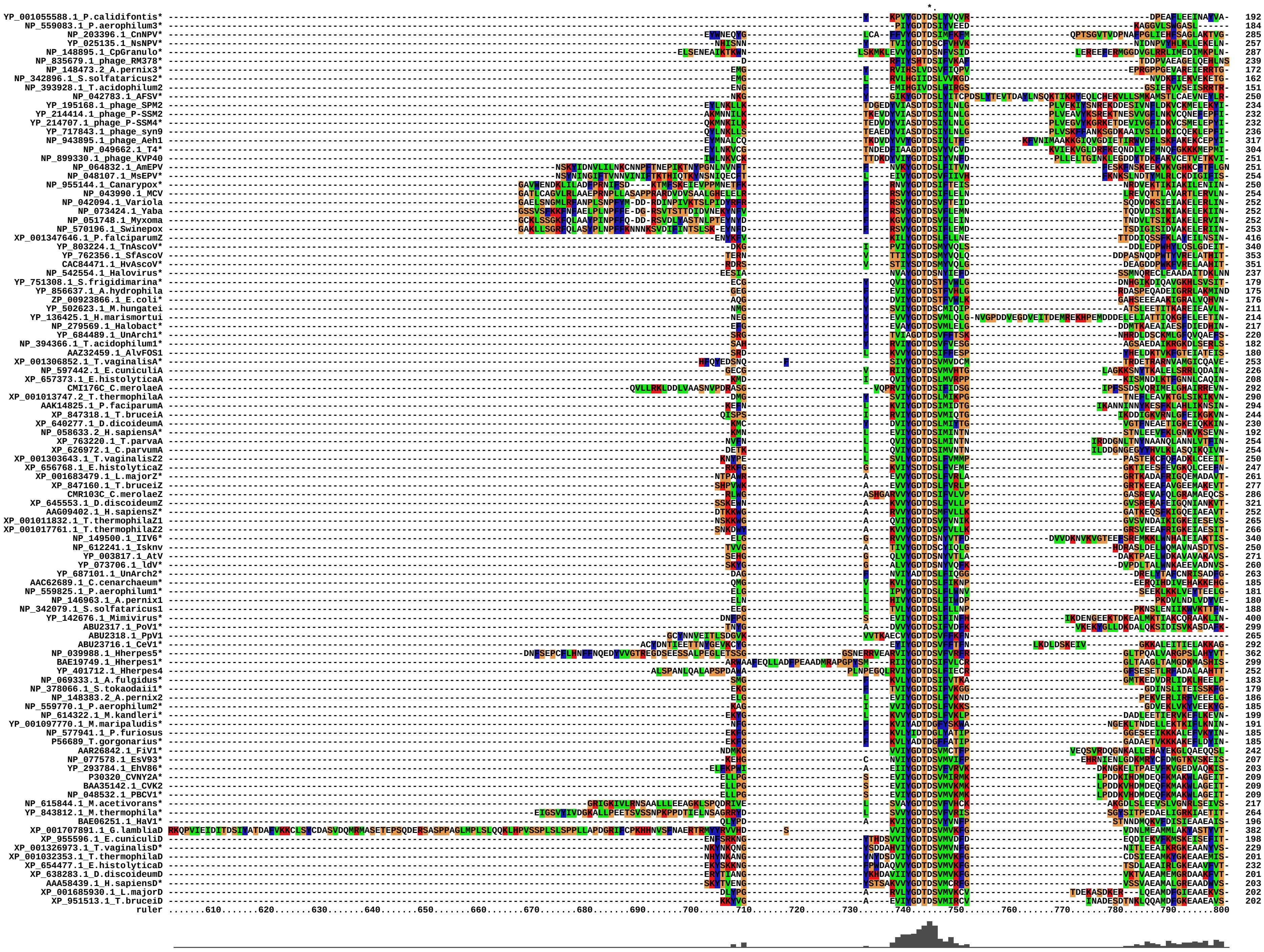

YP_001055588.1_P.calidifontis* 192
NP_559083.1_P.aerophilum3* 184
NP_203396.1_CnNPV* 285
YP_025135.1_NsNPV* 257
NP_148895.1_CpGranulo* 287
NP_835679.1_phage_RM378* 239
NP_148473.2_A.pernix3* 172
NP_342896.1_S.solfataricus2* 162
NP_393928.1_T.acidophilum2 151
NP_042783.1_AFSV* 250
YP_195168.1_phage_SPM2 234
YP_214414.1_phage_P-SSM2 232
YP_214707.1_phage_P-SSM4* 232
YP_717843.1_phage_syn9 236
NP_943895.1_phage_Aeh1 317
NP_049662.1_T4* 304
NP_899330.1_phage_KVP40 251
NP_064832.1_AmEPV 251
NP_048107.1_MsEPV* 254
NP_955144.1_Canarypox* 250
NP_043990.1_MCV 254
NP_042094.1_Variola 252
NP_073424.1_Yaba 252
NP_051748.1_Myxoma 252
NP_570196.1_Swinepox 253
XP_001347646.1_P.falciparumZ 416
YP_803224.1_TnAscoV* 340
YP_762356.1_SfAscoV 353
CAC84471.1_HvAscoV* 351
NP_542554.1_Halovirus* 237
YP_751308.1_S.frigidimarina* 179
YP_856637.1_A.hydrophila 175
ZP_00923866.1_E.coli* 176
YP_502623.1_M.hungatei 211
YP_136425.1_H.marismortui 214
NP_279569.1_Halobact* 217
YP_684489.1_UnArch1* 220
NP_394366.1_T.acidophilum1* 182
AAZ32459.1_AlvFOS1 180
XP_001306852.1_T.vaginalisA* 253
NP_597442.1_E.cuniculiA 226
XP_657373.1_E.histolyticaA 208
CMI176C_C.merolaeA 292
XP_001013747.2_T.thermophilaA 290
AAK14825.1_P.faciparumA 294
XP_847318.1_T.bruceiA 244
XP_640277.1_D.dicoideumA 230
NP_058633.2_H.sapiensA* 192
XP_763220.1_T.parvaA 254
XP_626972.1_C.parvumA 254
XP_001303643.1_T.vaginalisZ2 250
XP_656768.1_E.histolyticaZ 247
XP_001683479.1_L.majorZ* 261
XP_847160.1_T.bruceiZ 277
CMR103C_C.merolaeZ 286
XP_645553.1_D.discoideumZ 321
AAG09402.1_H.sapiensZ* 252
XP_001011832.1_T.thermophilaZ1 265
XP_001017761.1_T.thermophilaZ2 266
NP_149500.1_IIV6* 340
NP_612241.1_Isknv 250
YP_003817.1_AtV 271
YP_073706.1_ldV* 260
YP_687101.1_UnArch2* 263
AAC62689.1_C.cenarchaeum* 185
NP_559825.1_P.aerophilum1* 181
NP_146963.1_A.pernix1 180
NP_342079.1_S.solfataricus1 188
YP_142676.1_Mimivirus* 400
ABU2317.1_PoV1* 299
ABU2318.1_PpV1 265
ABU23716.1_CeV1* 292
NP_039988.1_Hherpes5* 362
BAE19749.1_Hherpes1* 299
YP_401712.1_Hherpes4 252
NP_069333.1_A.fulgidus* 183
NP_378066.1_S.tokadaii1* 179
NP_148383.2_A.pernix2 186
NP_559770.1_P.aerophilum2* 185
NP_614322.1_M.kandleri* 199
YP_001097770.1_M.maripaludis* 191
NP_577941.1_P.furiosus 185
P56689_T.gorgonarius* 185
AAR26842.1_FiV1* 242
NP_077578.1_EsV93* 207
YP_293784.1_EhV86* 203
P30320_CVNY2A* 209
BAA35142.1_CVK2 209
NP_048532.1_PBCV1* 209
NP_615844.1_M.acetivorans* 217
YP_843812.1_M.thermophila* 264
BAE06251.1_HaV1* 196
XP_001707891.1_G.lambliaD 382
XP_955596.1_E.cuniculiD 198
XP_001326973.1_T.vaginalisD* 229
XP_001032353.1_T.thermophilaD 201
XP_654477.1_E.histolyticaD 232
XP_638283.1_D.discoideumD 201
AAA58439.1_H.sapiensD* 203
XP_001685930.1_L.majorD 202
XP_951513.1_T.bruceiD 202
ruler ....610.......620.......630.......640.......650.......660.......670.......680.......690.......700.......710.......720.......730.......740.......750.......760.......770.......780.......790.......800

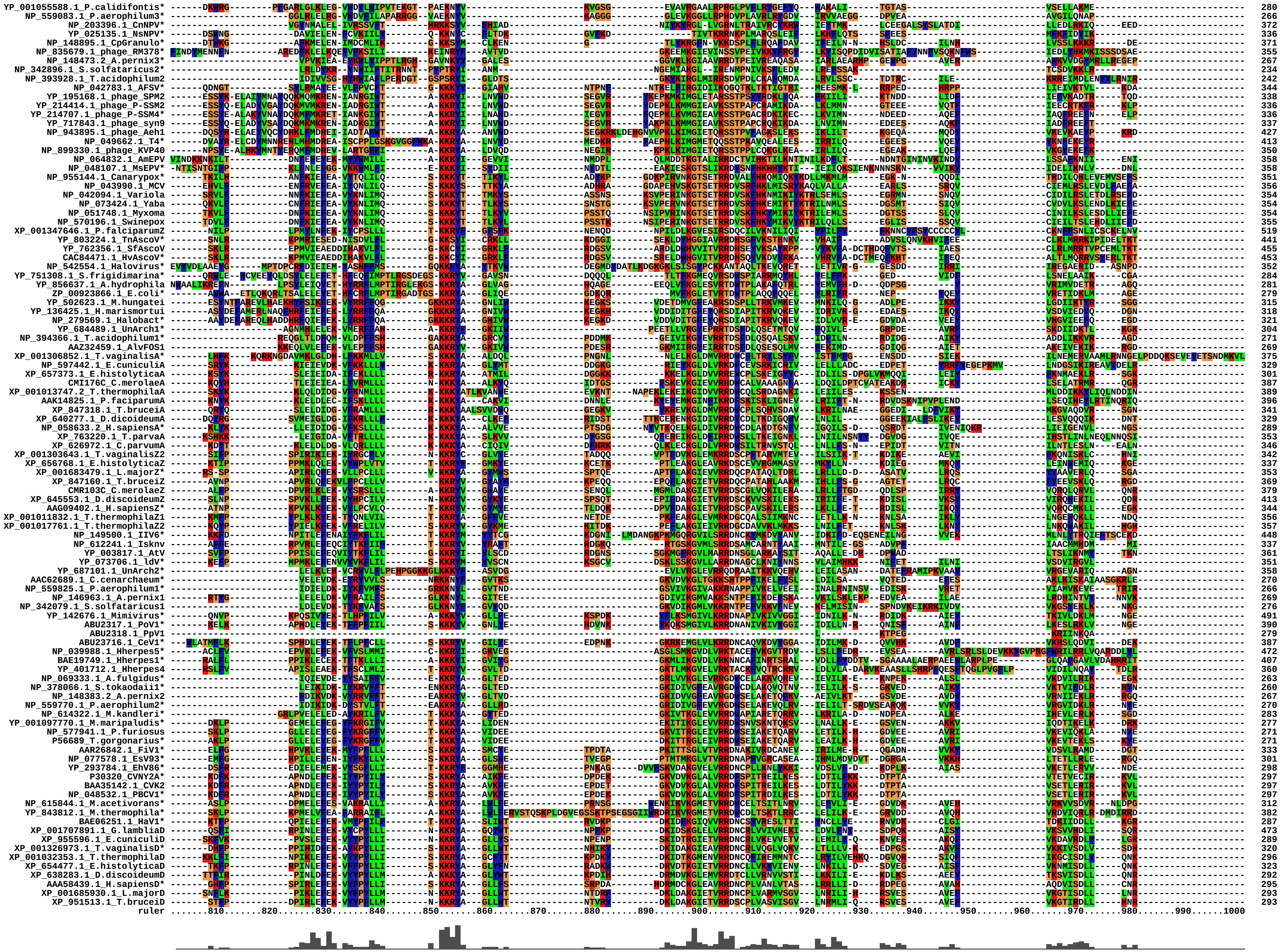
YP_001055588.1_P.calidifontis* 280
NP_559083.1_P.aerophilum3* 266
NP_203396.1_CnNPV* 372
YP_025135.1_NsNPV* 336
NP_148895.1_CpGranulo* 371
NP_835679.1_phage_RM378* 355
NP_148473.2_A.pernix3* 267
NP_342896.1_S.solfataricus2* 234
NP_393928.1_T.acidophilum2 242
NP_042783.1_AFSV* 344
YP_195168.1_phage_SPM2 338
YP_214414.1_phage_P-SSM2 336
YP_214707.1_phage_P-SSM4* 336
YP_717843.1_phage_syn9 337
NP_943895.1_phage_Aeh1 427
NP_049662.1_T4* 413
NP_899330.1_phage_KVP40 350
NP_064832.1_AmEPV 358
NP_048107.1_MsEPV* 358
NP_955144.1_Canarypox* 351
NP_043990.1_MCV 356
NP_042094.1_Variola 354
NP_073424.1_Yaba 354
NP_051748.1_Myxoma 354
NP_570196.1_Swinepox 355
XP_001347646.1_P.falciparumZ 519
YP_803224.1_TnAscoV* 441
YP_762356.1_SfAscoV 455
CAC84471.1_HvAscoV* 453
NP_542554.1_Halovirus* 352
YP_751308.1_S.frigidimarina* 284
YP_856637.1_A.hydrophila 281
ZP_00923866.1_E.coli* 279
YP_502623.1_M.hungatei 315
YP_136425.1_H.marismortui 318
NP_279569.1_Halobact* 321
YP_684489.1_UnArch1* 304
NP_394366.1_T.acidophilum1* 271
AAZ32459.1_AlvFOS1 269
XP_001306852.1_T.vaginalisA* 375
NP_597442.1_E.cuniculiA 329
XP_657373.1_E.histolyticaA 301
CMI176C_C.merolaeA 387
XP_001013747.2_T.thermophilaA 389
AAK14825.1_P.faciparumA 396
XP_847318.1_T.bruceiA 341
XP_640277.1_D.dicoideumA 329
NP_058633.2_H.sapiensA* 289
XP_763220.1_T.parvaA 353
XP_626972.1_C.parvumA 346
XP_001303643.1_T.vaginalisZ2 342
XP_656768.1_E.histolyticaZ 337
XP_001683479.1_L.majorZ* 353
XP_847160.1_T.bruceiZ 369
CMR103C_C.merolaeZ 379
XP_645553.1_D.discoideumZ 413
AAG09402.1_H.sapiensZ* 344
XP_001011832.1_T.thermophilaZ1 356
XP_001017761.1_T.thermophilaZ2 357
NP_149500.1_IIV6* 448
NP_612241.1_Isknv 337
YP_003817.1_AtV 361
YP_073706.1_ldV* 351
YP_687101.1_UnArch2* 358
AAC62689.1_C.cenarchaeum* 270
NP_559825.1_P.aerophilum1* 266
NP_146963.1_A.pernix1 269
NP_342079.1_S.solfataricus1 276
YP_142676.1_Mimivirus* 491
ABU2317.1_PoV1* 390
ABU2318.1_PpV1 279
ABU23716.1_CeV1* 387
NP_039988.1_Hherpes5* 472
BAE19749.1_Hherpes1* 407
YP_401712.1_Hherpes4 360
NP_069333.1_A.fulgidus* 263
NP_378066.1_S.tokadaii1* 260
NP_148383.2_A.pernix2 267
NP_559770.1_P.aerophilum2* 270
NP_614322.1_M.kandleri* 283
YP_001097770.1_M.maripaludis* 277
NP_577941.1_P.furiosus 271
P56689_T.gorgonarius* 271
AAR26842.1_FiV1* 333
NP_077578.1_EsV93* 301
YP_293784.1_EhV86* 298
P30320_CVNY2A* 297
BAA35142.1_CVK2 297
NP_048532.1_PBCV1* 297
NP_615844.1_M.acetivorans* 312
YP_843812.1_M.thermophila* 382
BAE06251.1_HaV1* 287
XP_001707891.1_G.lambliaD 473
XP_955596.1_E.cuniculiD 289
XP_001326973.1_T.vaginalisD* 320
XP_001032353.1_T.thermophilaD 296
XP_654477.1_E.histolyticaD 323
XP_638283.1_D.discoideumD 292
AAA58439.1_H.sapiensD* 295
XP_001685930.1_L.majorD 293
XP_951513.1_T.bruceiD 293
ruler ....810.......820.......830.......840.......850.......860.......870.......880.......890.......900.......910.......920.......930.......940.......950.......960.......970.......980.......990......1000

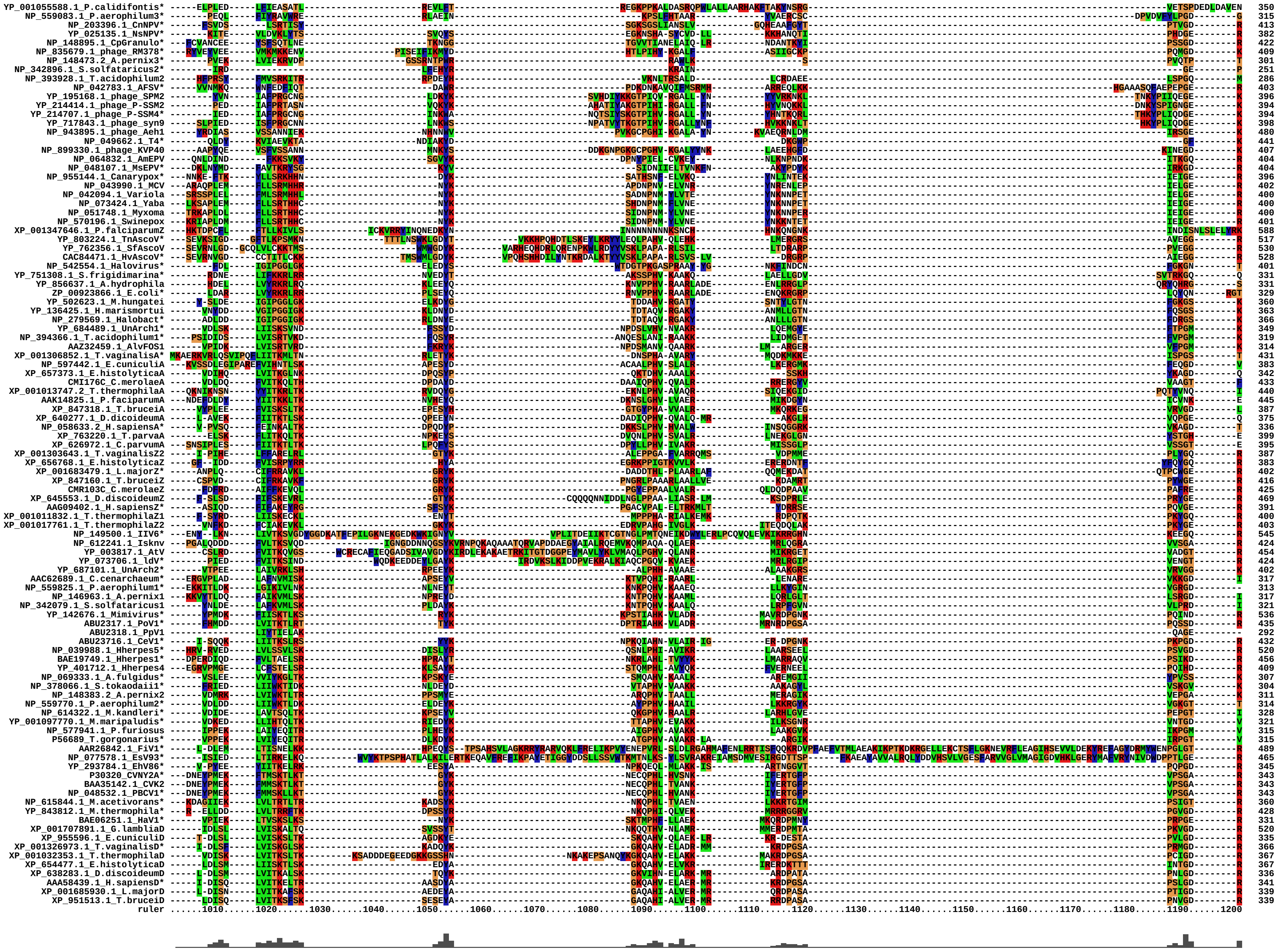
YP_001055588.1_P.calidifontis*
NP_559083.1_P.aerophilum3*
NP_203396.1_CnNPV*
YP_025135.1_NsNPV*
NP_148895.1_CpGranulo*
NP_835679.1_phage_RM378*
NP_148473.2_A.pernix3*
NP_342896.1_S.solfataricus2*
NP_393928.1_T.acidophilum2
NP_042783.1_AFSV*
YP_195168.1_phage_SPM2
YP_214414.1_phage_P-SSM2
YP_214707.1_phage_P-SSM4*
YP_717843.1_phage_syn9
NP_943895.1_phage_Aeh1
NP_049662.1_T4*
NP_899330.1_phage_KVP40
NP_064832.1_AmEPV
NP_048107.1_MsEPV*
NP_955144.1_Canarypox*
NP_043990.1_MCV
NP_042094.1_Variola
NP_073424.1_Yaba
NP_051748.1_Myxoma
NP_570196.1_Swinepox
XP_001347646.1_P.falciparumZ
YP_803224.1_TnAscoV*
YP_762356.1_SfAscoV
CAC84471.1_HvAscoV*
NP_542554.1_Halovirus*
YP_751308.1_S.frigidimarina*
YP_856637.1_A.hydrophila
ZP_00923866.1_E.coli*
YP_502623.1_M.hungatei
YP_136425.1_H.marismortui
NP_279569.1_Halobact*
YP_684489.1_UnArch1*
NP_394366.1_T.acidophilum1*
AAZ32459.1_AlvFOS1
XP_001306852.1_T.vaginalisA*
NP_597442.1_E.cuniculiA
XP_657373.1_E.histolyticaA
CMI176C_C.merolaeA
XP_001013747.2_T.thermophilaA
AAK14825.1_P.faciparumA
XP_847318.1_T.bruceiA
XP_640277.1_D.dicoideumA
NP_058633.2_H.sapiensA*
XP_763220.1_T.parvaA
XP_626972.1_C.parvumA
XP_001303643.1_T.vaginalisZ2
XP_656768.1_E.histolyticaZ
XP_001683479.1_L.majorZ*
XP_847160.1_T.bruceiZ
CMR103C_C.merolaeZ
XP_645553.1_D.discoideumZ
AAG09402.1_H.sapiensZ*
XP_001011832.1_T.thermophilaZ1
XP_001017761.1_T.thermophilaZ2
NP_149500.1_IIV6*
NP_612241.1_Isknv
YP_003817.1_AtV
YP_073706.1_ldV*
YP_687101.1_UnArch2*
AAC62689.1_C.cenarchaeum*
NP_559825.1_P.aerophilum1*
NP_146963.1_A.pernix1
NP_342079.1_S.solfataricus1
YP_142676.1_Mimivirus*
ABU2317.1_PoV1*
ABU2318.1_PpV1
ABU23716.1_CeV1*
NP_039988.1_Hherpes5*
BAE19749.1_Hherpes1*
YP_401712.1_Hherpes4
NP_069333.1_A.fulgidus*
NP_378066.1_S.tokodaii1*
NP_148383.2_A.pernix2
NP_559770.1_P.aerophilum2*
NP_614322.1_M.kandleri*
YP_001097770.1_M.maripaludis*
NP_577941.1_P.furiosus
P56689_T.gorgonarius*
AAR26842.1_FiV1*
NP_077578.1_EsV93*
YP_293784.1_EhV86*
P30320_CVNY2A*
BAA35142.1_CVK2
NP_048532.1_PBCV1*
NP_615844.1_M.acetivorans*
YP_843812.1_M.thermophila*
BAE06251.1_HaV1*
XP_001707891.1_G.lambliaD
XP_955596.1_E.cuniculiD
XP_001326973.1_T.vaginalisD*
XP_001032353.1_T.thermophilaD
XP_654477.1_E.histolyticaD
XP_638283.1_D.discoideumD
AAA58439.1_H.sapiensD*
XP_001685930.1_L.majorD
XP_951513.1_T.bruceiD
ruler

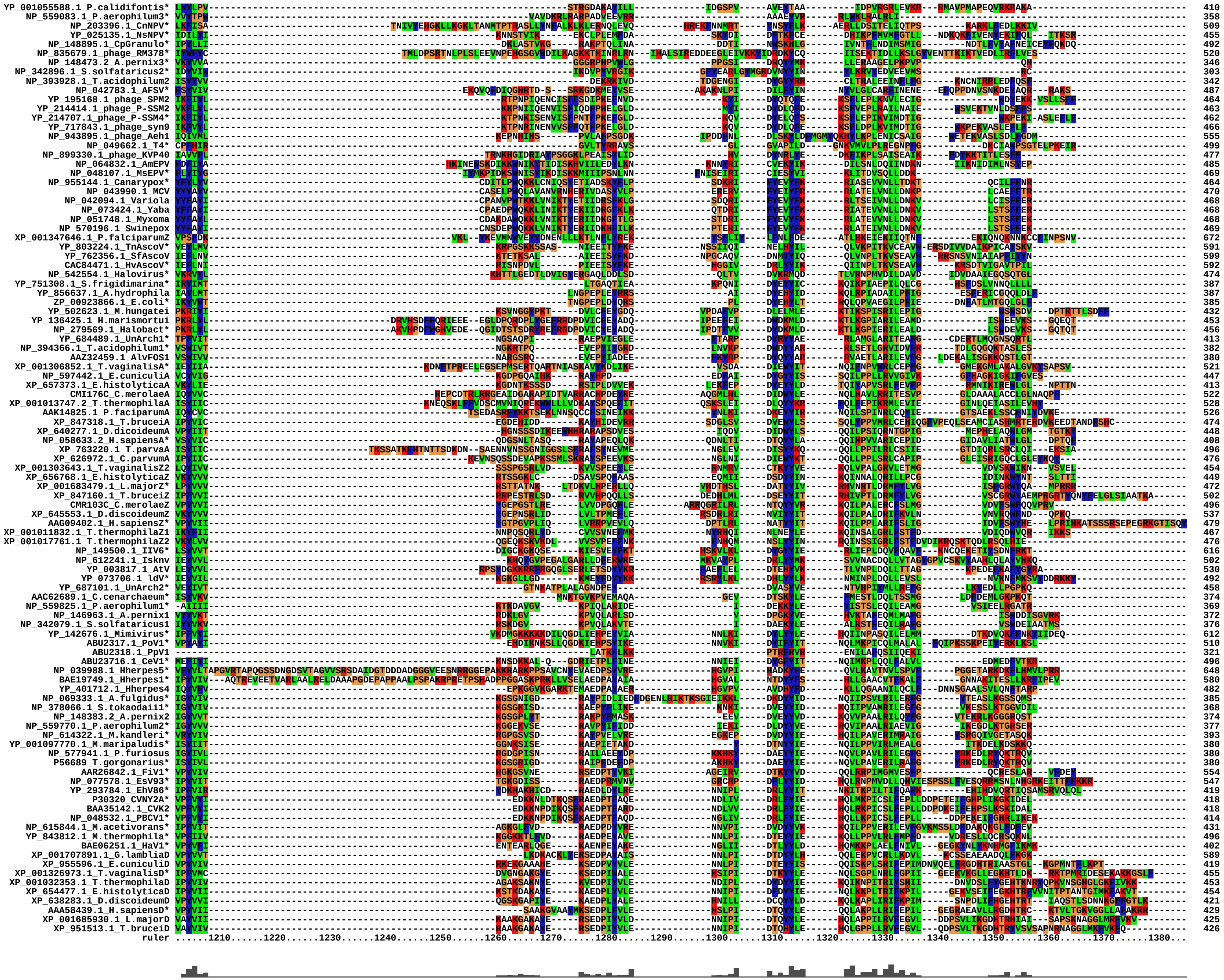

Supplement: Additional data file 2 — Sequences used in the final reduced reference multiple alignment are displayed with an asterisk. [file gb-2008-9-7-r106-S2.pdf]
