# Supplementary figures and images for: Taxonomic distribution of large DNA viruses in the sea
Source: Genome Biol. 2008 Jul 3;9(7):R106. doi: 10.1186/gb-2008-9-7-r106 (PMC2530865; doi:10.1186/gb-2008-9-7-r106)

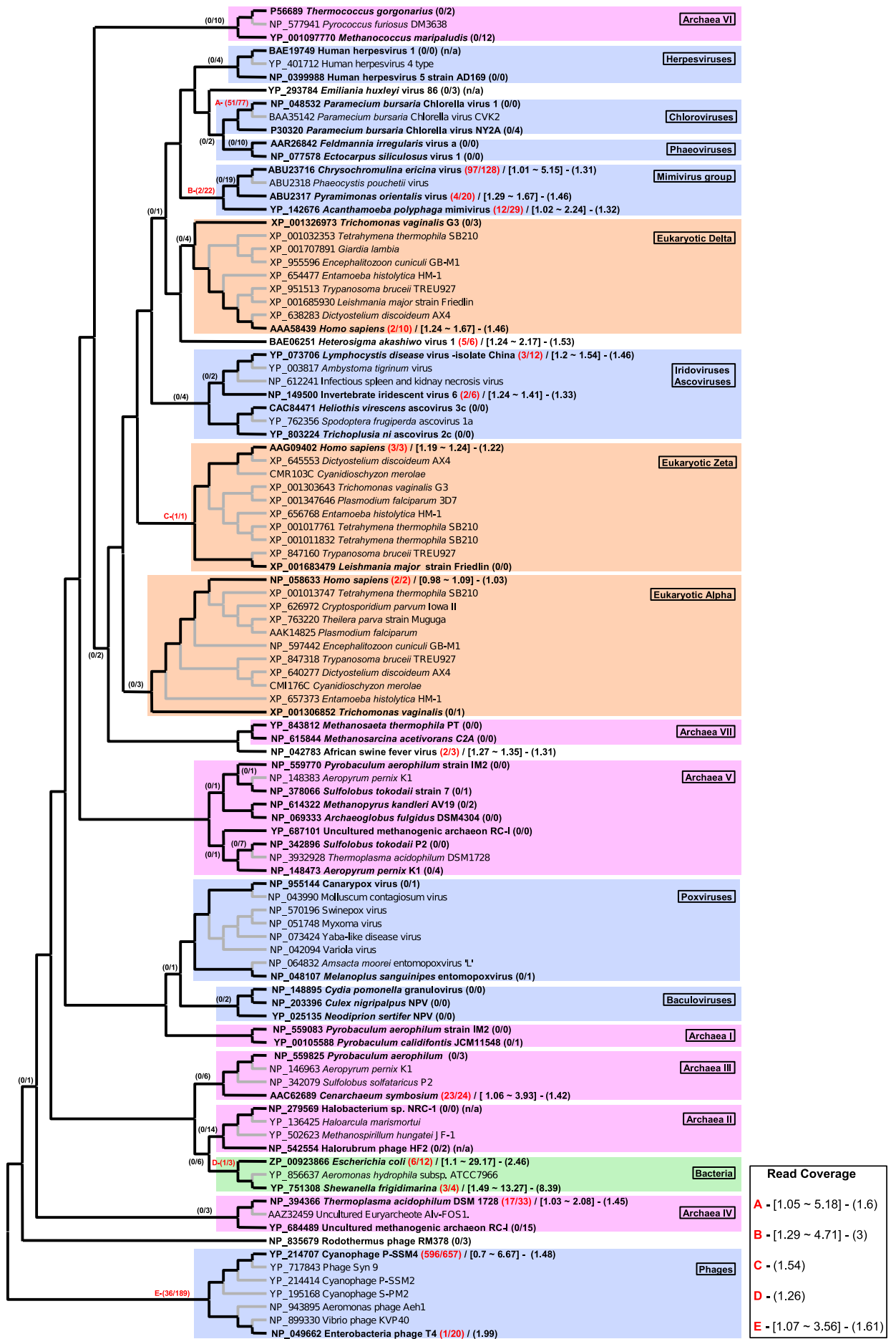

Supplement: Additional data file 3 — The GOS PolB fragments are displayed for each of the 99 branches tested. Numbers in parentheses (V/W) are the total number of mapped PolB fragments (W) and the number of supported cases (V) (displayed in red). Read coverage values are presented as follows: [X-Y]-(Z) where X and Y are the read coverage value range (minimum/maximum) and Z the read coverage median value. [file gb-2008-9-7-r106-S3.pdf]
